# Shaping Neuronal Network Activity by Presynaptic Mechanisms

**DOI:** 10.1371/journal.pcbi.1004438

**Published:** 2015-09-15

**Authors:** Ayal Lavi, Omri Perez, Uri Ashery

**Affiliations:** 1 Department of Neurobiology, Life Sciences Institute, Tel Aviv University, Tel Aviv, Israel; 2 Sagol School of Neuroscience, Tel Aviv University, Tel Aviv, Israel; 3 Sackler School of Medicine, Tel Aviv University, Tel Aviv, Israel; École Normale Supérieure, College de France, CNRS, FRANCE

## Abstract

Neuronal microcircuits generate oscillatory activity, which has been linked to basic functions such as sleep, learning and sensorimotor gating. Although synaptic release processes are well known for their ability to shape the interaction between neurons in microcircuits, most computational models do not simulate the synaptic transmission process directly and hence cannot explain how changes in synaptic parameters alter neuronal network activity. In this paper, we present a novel neuronal network model that incorporates presynaptic release mechanisms, such as vesicle pool dynamics and calcium-dependent release probability, to model the spontaneous activity of neuronal networks. The model, which is based on modified leaky integrate-and-fire neurons, generates spontaneous network activity patterns, which are similar to experimental data and robust under changes in the model's primary gain parameters such as excitatory postsynaptic potential and connectivity ratio. Furthermore, it reliably recreates experimental findings and provides mechanistic explanations for data obtained from microelectrode array recordings, such as network burst termination and the effects of pharmacological and genetic manipulations. The model demonstrates how elevated asynchronous release, but not spontaneous release, synchronizes neuronal network activity and reveals that asynchronous release enhances utilization of the recycling vesicle pool to induce the network effect. The model further predicts a positive correlation between vesicle priming at the single-neuron level and burst frequency at the network level; this prediction is supported by experimental findings. Thus, the model is utilized to reveal how synaptic release processes at the neuronal level govern activity patterns and synchronization at the network level.

## Author Summary

The activity of neuronal networks underlies basic neural functions such as sleep, learning and sensorimotor gating. Computational models of neuronal networks have been developed to capture the complexity of the network activity and predict how neuronal networks generate spontaneous activity. However, most computational models do not simulate the intricate synaptic release process that governs the interaction between neurons and has been shown to significantly impact neuronal network activity and animal behavior, learning and memory. Our paper demonstrates the importance of simulating the elaborate synaptic release process to understand how neuronal networks generate spontaneous activity and respond to manipulations of the release process. The model provides mechanistic explanations and predictions for experimental pharmacological and genetic manipulations. Thus, the model presents a novel computational platform to understand how mechanistic changes in the synaptic release process modulate network oscillatory activity that might impact basic neural functions.

## Introduction

Oscillatory activity patterns in the brain have been linked to sleep, sensorimotor gating, short-term memory storage and selective attention [1,2]. Neuronal microcircuits in the brain spontaneously generate oscillatory activity patterns via synaptic interaction between groups of neurons [1,2]. Indeed, changes in synaptic transmission cause alterations in neuronal firing and neuronal network activity [3–5], and synaptic dysfunction can lead to pathological epileptic conditions [6–8]. Even though small alterations in synaptic transmission and in the firing properties of single neurons can alter the spontaneous and evoked activity of entire neuronal circuits [3,9], most computational models of neuronal networks do not explicitly account for the elaborate presynaptic neurotransmission process.

Presynaptic transmission is a regulated multistep process that encompasses the loading of neurotransmitters into synaptic vesicles, the translocation to and docking of those vesicles at the plasma membrane (PM), and vesicle preparation for fusion through a calcium-dependent maturation process generally referred to as "vesicle priming" [10–14]. This pool of primed vesicles is the readily releasable pool (RRP), where vesicles undergo immediate fusion with the PM upon acute elevation in intracellular calcium concentration ([Ca^2+^]_i_). Another presynaptic pool of vesicles, the recycling pool (ReP), accommodates unprimed vesicles which can undergo maturation and fusion during repetitive synaptic stimulation; all of the remaining vesicles in the presynaptic terminal belong to the reserve pool (RP).

Equilibrium of the presynaptic vesicles transition between these pools depends on neuronal activity, synaptic proteins and calcium [15–19]. In the synapses, there are three types of synaptic release modes that rely on the high dynamic range of [Ca^2+^]_i_ and share the same vesicle pools [20,21] (but see [22,23]). They are defined by their temporal association with the action potential (AP): a) synchronous release, driven by a short-lived acute increase in [Ca^2+^]_i_, is time-locked to the AP [24–26]; b) asynchronous release begins several milliseconds after an AP and drives slower vesicle release; this rate is two orders of magnitude slower than that of synchronous release. Asynchronous release is enhanced by slow clearance of residual calcium from the presynaptic terminal, as well as by strontium application [24]; c) spontaneous release which emerges without any association to previous neuronal activity. Although presynaptic transmission is well understood at the single-neuron level, it is unclear how the aforedescribed manipulation of presynaptic processes modulates the activity patterns and synchronization of the network. Recently, a handful of studies have begun to investigate how manipulations of non-synchronous presynaptic release, such as asynchronous or spontaneous release, modulate neuronal network activity [3,6–8,24,25,27–30].

Understanding the determinant properties of spontaneous activity of the neuronal network is highly complex. Therefore, neuronal network computational models are utilized to simulate key features of the network's spontaneous activity. A large group of simulations utilizes computationally light leaky integrate-and-fire (LIF) neurons to model the activity of large-scale neuronal networks [31,32]. However, these neuronal models are based on very general assumptions regarding neuronal synaptic transmission processes and thus do not simulate critical synaptic mechanisms, such as the transition of vesicles between pools, vesicle maturation steps or calcium-dependent presynaptic release. An important model for neuronal networks, which combines the concept of synaptic resources and neuronal activity, is the tri-state model [33]. The original model, based on three kinetic equations, organized synaptic resources into three states: active, recovered or inactive. Synaptic transmission in this model was determined by the available synaptic resources (i.e. vesicles) and a constant utilization factor (i.e. calcium, according to the calcium-based synaptic release theory). This model was later extended to include an increase in the utilization factor as the neuron keeps firing [34–36], much like the increase in [Ca^2+^]_i_ occurring in short-term synaptic plasticity. Another extension of the model also included asynchronous synaptic transmission by adding a super-inactive state [29,30,37,38] to generate reverberatory activity in small networks. Nonetheless, this model does not directly simulate the presynaptic vesicle pools, calcium-dependent vesicle priming or calcium-dependent release, which are basic and crucial properties of presynaptic release [25,39–42]. Furthermore, in response to evoked stimulations, this model generates very short network oscillations (each oscillation lasting several milliseconds), which are several orders of magnitude shorter and more frequent than the network bursts recorded *in vitro* (typically several hundreds of milliseconds of recurrent network activity) [43,44]. In general, none of these models simulate spontaneous release, which is physiologically important [45–47], and spontaneous activity in these models is generally achieved by artificial injection of current [48–50]. In addition, a model that relates in detail to changes in synaptic processes, and provides a mechanistic explanation and prediction for how changes in synaptic mechanisms at the neuronal level govern the activity patterns and synchronization at the network level is lacking.

In this paper, we present a novel computational model that demonstrates how changes in synaptic transmission modulate neuronal network activity patterns. We utilized experimental data from *in vitro* neuronal networks cultured on microelectrode arrays (MEA) that spontaneously generate network-wide synchronized activity patterns, termed network bursts. We used the model to learn about synaptic mechanisms that can explain changes in neuronal network activity following manipulations of the presynaptic release processes [3,5,43,44,51]. Our model attempts to strike a balance between detailed cellular models and simplified neuronal network models [15,21,26,52] by extending the LIF neuronal model to simulate both the presynaptic release process and the entire neuronal network. This allowed us to examine how manipulations of specific steps in the presynaptic release mechanism affect neuronal network activity. The model provides putative mechanistic explanations for various network activity patterns *in vitro*, such as network burst termination, and allows us to predict how changes in the presynaptic release machinery will affect network oscillation frequency.

## Results

### Integrating the presynaptic release mechanism into a neuronal network model

**Fig 1:**
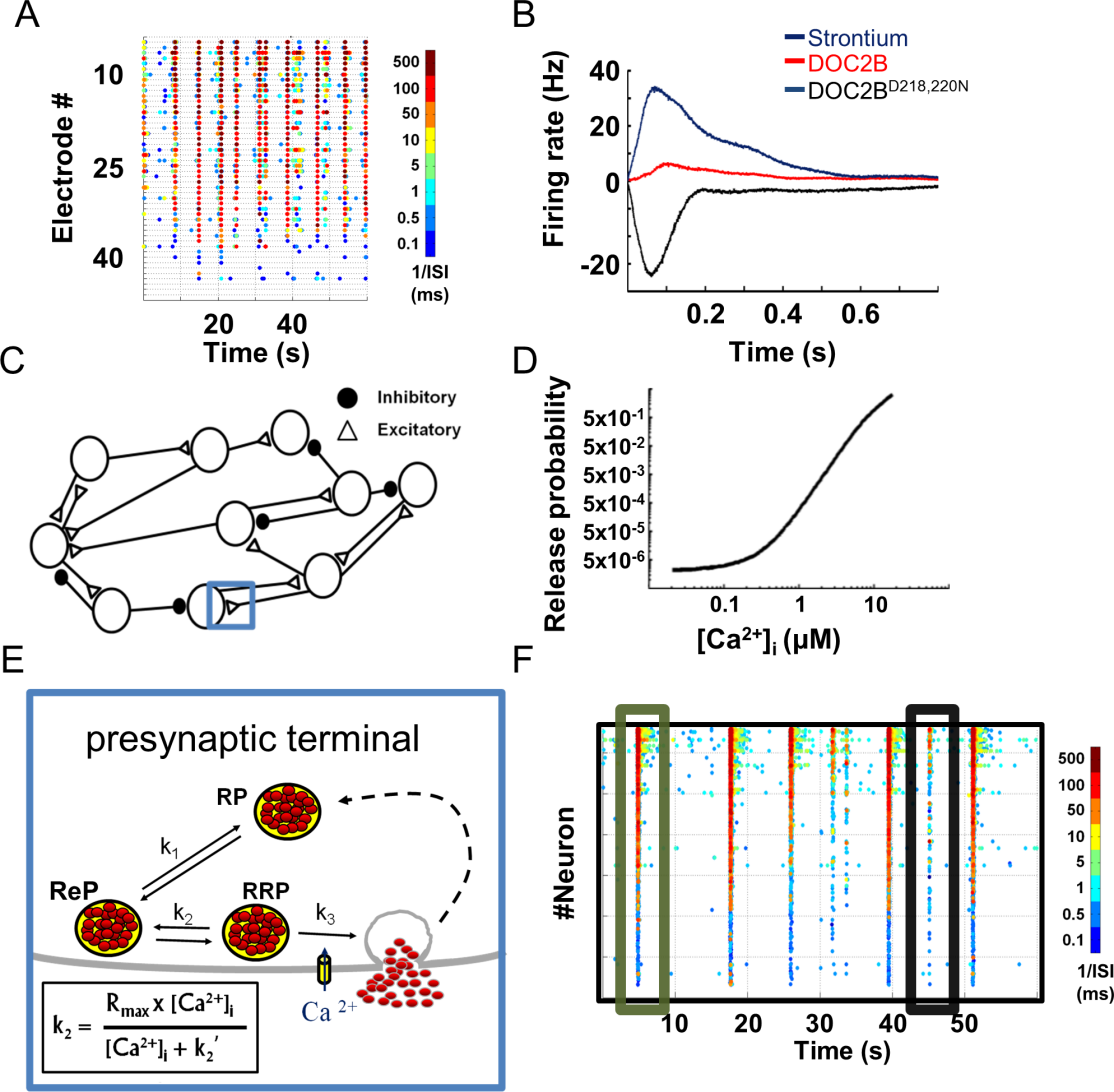
Presynaptic-driven neuronal network computational model recreates spontaneous network activity recorded with microelectrode arrays. **(A)** Color-coded raster plots of spontaneous neuronal network activity recorded on microelectrode array (each dot denotes spike from an electrode, colors code for average firing rate). **(B)** Profiles of neuronal network bursts demonstrating increased average firing rate with strontium application and DOC2B expression; DOC2B mutant (DOC2B^D218,220N^) reduces network burst firing rate (modified from Lavi et al. [3]). **(C)** Computational model comprises a network of excitatory and inhibitory neurons. Each connection/synapse accommodates a multistep process, as detailed in E (one synapse is marked with a blue rectangle and its synaptic components are detailed in E). **(D)** Synaptic release probability in the model is a function of intracellular calcium concentration ([Ca^2+^]_i_). **(E)** Each synapse comprises reserve (RP), recycling (ReP) and readily releasable (RRP) vesicle pools. The transition between pools is bidirectional and is determined by the k_i_ rate constants (k_2_ is Ca^2+^-dependent). **(F)** Spontaneous activity generated by the model is very similar to the experimental recording shown in A (each dot denotes neuronal action potential, colors code for average firing rate). ISI, inter-spike interval.

We previously explored [3] how genetic and pharmacological manipulations of presynaptic release change the spontaneous activity of neuronal networks cultured on MEA plates (Figs 1A and S1). To do so, we genetically and pharmacologically manipulated different synaptic transmission steps in cultured neuronal networks and examined the effects on neuronal network activity. Pharmacological enhancement of asynchronous release was achieved by strontium application, which has been shown to activate calcium-dependent release mechanisms but is cleared from the presynaptic terminal more slowly than calcium [53,54]. Genetic manipulations consisted of overexpressing DOC2B, a presynaptic protein that enhances spontaneous and asynchronous neurotransmitter release [55–57]. Our findings suggested that higher levels of asynchronous release at single synapses, induced by DOC2B overexpression or by strontium application, increase the firing rate within a network burst; on the other hand, facilitation of spontaneous release frequency by overexpression of the DOC2B^D218,220N^ mutant [3] led to lower network burst firing rate (Figs 1B and S1). These findings join other studies that have shown that manipulation of presynaptic proteins has a substantial impact on neuronal network plasticity, information transfer and animal behavior [10,58,59]. However, it is difficult to infer a mechanistic explanation for these findings. Therefore, we developed a computational model that simulates how changes in different steps of synaptic transmission alter neuronal firing.

The model consisted of 800 LIF neurons, spread on a virtual MEA-like 2D surface (30% inhibitory neurons; Fig 1C) [3]. The neurons were connected by the small-world and scale-free topology typically associated with cortical neuronal networks [60–63] (S2 Fig), creating an active neuronal network. A key feature of the model was that neuronal activity and synaptic release were generated from a presynaptic compartment that simulates the multistep process of calcium-dependent synaptic transmission. This presynaptic compartment was simulated for each LIF neuron (Eq 1) and governed the spontaneous, evoked and asynchronous activity of each neuron in the network. All of the chosen parameters were based on up-to-date papers (Table 1) [63]. Our model allowed us to perform *in silico* experiments, manipulate specific properties of synaptic transmission and study their impact at the network level. It gave us access to multiple cellular parameters, such as vesicular pool capacities, vesicle replenishment rate and [Ca^2+^]_i_, and simultaneously follow the macroscale network activity and the interaction between neurons.

**Table 1:** Summary of the primary parameters of the neuronal network model.

	Parameter	Value	Description
**General**			
	MEA_Dims	100x100	MEA dimensions in arbitrary units
	N	800	Number of neurons
	Connectivity_ratio	5%	Ratio of actual connections out of all possible connections
	inhibitory_neuron_ratio	30%	Ratio of inhibitory neurons
	Weight_params_mu **(μ** ^**Aij**^ **)**	-0.874	Synapse lognormal weight mean [96]
	Weight_params_sigma **(σ** ^**Aij**^ **)**	1.026	Synapse lognormal weight standard deviation [96]
**Voltage**			
	average_EPSP	3.16	**mV**; average postsynaptic potential [98]
	standard_voltage	-70	**mV**; resting potential
	max_voltage	50	**mV**; maximum voltage after action potential (AP)
	hyper_polarize_voltage **(V**_**hyp**_ **)**	-77	**mV**; voltage after hyperpolarization
	AP_threshold **(θ)**	-30	**mV**; threshold for AP generation
	voltage_tau **(τ**_**m**_ **)**	52	**ms**; membrane voltage decay rate *(measured experimentally)*
	refractory_period **(τ**_**arp**_ **)**	3	**ms**; refractory period after AP
**Vesicle pools**			
	standard_RRP **(RRP**_**full**_ **)**	10	Number of vesicles in readily releasable pool (RRP) [97]
	standard_ReP **(ReP**_**full**_ **)**	20	Number of vesicles in recycling pool (ReP) [97]
	standard_RP **(RP_*full*_)**	170	Number of vesicles in reserve pool (RP) [97]
	single_vesicle_max_RRP_repleneshing_k **(R**_**max**_ **)**	7.3e-4	**s** ^**-1**^; maximum single-vesicle transfer rate from ReP to RRP [67]
	ReP_repleneshing_tau **(*τ*_*RP*→*ReP*_)**	30000	**ms**; ReP replenishing rate [15]
	RP_repleneshing_tau **(*τ*_→*RP*_)**	50000	**ms**; RP replenishing rate
**Calcium**			
	fast_calcium_max **(CA**_**FAST**_ **)**	13.6	**μM**; maximum calcium concentration in fast calcium [25]
	slow_calcium_max **(CA**_**SLOW**_ **)**	1.36	**μM**; maximum calcium concentration in slow calcium [25]
	slow_calcium_AP_influx	0.5	**μM**; calcium increment for each AP [99,100]
	calcium_fast_efflux_tau **(*τ*_*Ca*_*fast*__)**	1	**ms**; fast calcium efflux rate [25]
	calcium_slow_efflux_tau **(*τ*_*Ca*_*slow*__)**	31	**ms**; slow calcium efflux rate [99]
	basal_calcium	0.05	**μM**; basal calcium concentration
	Kd	2.3	**μM**; dissociation constant affecting RRP refilling [67]
	p_max	0.15	Maximum release probability at maximum calcium [101,102]
	p_basal_perRRPpool	7.5e-4	Basal release probability at basal calcium [25]
	calcium_release_A **(α)**	0.175	Calcium-dependent release probability curve [25]
	calcium_release_B **(β)**	2.35	Calcium-dependent release probability curve [25]
	calcium_release_C **(γ)**	0.78	Calcium-dependent release probability curve [25]
	calcium_release_D **(δ)**	-3.6e-3	Calcium-dependent release probability curve [25]

The table summarizes the primary parameters used to construct and run the neuronal network computational model under baseline conditions. It also includes references to the original papers [15,25,67,96–101]. Parameter names correspond to the variable names used in the MATLAB code.

Each neuron received multiple inputs which accumulated as changes in the PM voltage until they crossed a threshold (Eq 2) and generated an AP or decayed with a predefined time constant (Table 1). AP generation induced a transient increase in the [Ca^2+^]_i_ that accumulates when several APs arrive concomitantly (Eq 4). This increase in calcium was then translated into vesicle release according to a calcium-dependent synaptic release curve (Fig 1D). The release curve (described in Eq 5) linked the free synaptic [Ca^2+^]_i_ to synaptic release probability (Pr) according to well-established release-rate curves [21,25,26]. According to most calcium-dependent release models, upon AP generation, calcium level increases by almost two to four orders of magnitude in the active zone, inducing an acute shift in the synaptic Pr [25,26]. Accordingly, we used the Calyx of Held calcium-dependent release-rate curve as previously described [26] with a small modification to fit the lower Pr of cortical synapses.

To recreate the multiscale temporal dynamics of synaptic release, each synapse consisted of three vesicle pools: RP (170 vesicles), ReP (20 vesicles) and RRP (10 vesicles) (Fig 1E); the vesicle transportation between pools was bidirectional (Eqs 7 and 8). Following vesicle release, vesicles underwent refilling according to different rate constants (Table 1). A variety of neuronal preparations have demonstrated that vesicle recruitment in neurons is enhanced by elevated [Ca^2+^]_i_ [41,64,65], and this enhancement has been recognized as essential for maintaining adequate release during high-frequency bursts of activity [65,66]. Therefore, we adapted the rate of vesicle transition from the ReP to the RRP to a similar Michaelis–Menten-type equation (Fig 1E black frame; Eq 7) which has been used to describe the calcium-dependent transition rate from the unprimed pool to the RRP in chromaffin cells [15,67]. Each vesicle fusion event contributes a positive or negative voltage upon release (excitatory or inhibitory postsynaptic potential, respectively) to the PM of the postsynaptic neuron. Notably, the basal activity in the model was maintained by spontaneous release driven from the Pr of the neuron under resting calcium levels (Eq 5). This method kept the network active and replaced the common route of keeping computational neuronal networks spontaneously active, i.e. injecting current into the neurons [48–50]. Comparison of network spontaneous activity between these two methods showed that calcium-dependent synaptic release generates network bursts which are more similar to those recorded from neuronal networks cultured on MEA (S3 Fig).

Recurrent network-wide bursting activity and abundant inter-burst activity can be observed in the color-coded raster plot of neuronal network spontaneous activity generated by the model (Fig 1F). Hence, the model recreated a pattern of synchronized activity followed by a period of quiescence similar to that in the experimental recordings (compare Fig 1F to 1A). Importantly, the model recreated both network-wide bursting activity ("full" bursts; green box) and bursting activity limited to subnetworks ("aborted" bursts; black box).

To test the stability and robustness of the network activity under various manipulations, we explored the response of the model to changes in its primary gain parameters: excitatory postsynaptic potential (EPSP, voltage) and connectivity ratio (the percentage of actual connections out of all possible connections in the network; see considerations for choosing these parameters in Methods). Quantitative analysis of the basic model activity parameters, such as global and network burst spike rate, network burst frequency and network burst duration, was performed under different levels of the gain parameters (S4 Fig). We found that the model is robust to two- to threefold changes in basic gain parameters while maintaining continuous spontaneous network activity but displaying changes in various network activity properties (S4 Fig). We also showed that even increasing the number of neurons or the number of synapses in the model 10-fold does not change its basic bursting activity; the neuronal network still displayed network-wide bursts followed by periods of relative quiescence (S5 Fig). The stability of the bursting activity of the network following changes in basic gain parameters (and changes in the number of neurons and number of independent synapses per neuron) established the robustness of the model and increased its fidelity. Indeed, most of the experimental manipulations did not abolish the basic bursting activity in the network but rather manipulated the inter-burst and intra-burst spiking profiles. This places the model in an excellent position to test the impact of changes in other parameters of synaptic release on the network bursting activity.

### Asynchronous release shapes network burst activity

The established model was utilized to understand two intriguing findings: elevated asynchronous release, but not spontaneous release, at the single-neuron level enhances and synchronizes network burst activity [3]; on the other hand, enhanced spontaneous release reduces synchronization and network burst activity. Experimentally, asynchronous release was elevated by either DOC2B or strontium. Strontium has been suggested to trigger vesicle fusion and neurotransmitter release in the same way as calcium, but is extruded from the synapse more slowly than calcium, causing long-lasting vesicle fusion or asynchronous neurotransmitter release [53,68]. Therefore, to mimic the effect of asynchronous release, we reduced the rate of calcium efflux out of the synapse (Eq 4, *τ*_*Ca*_*fast*__ and *τ*_*Ca*_*slow*__), allowing more time for vesicle fusion [69]. It is important to note that we changed the asynchronous release in both excitatory and inhibitory neurons.

**Fig 2:**
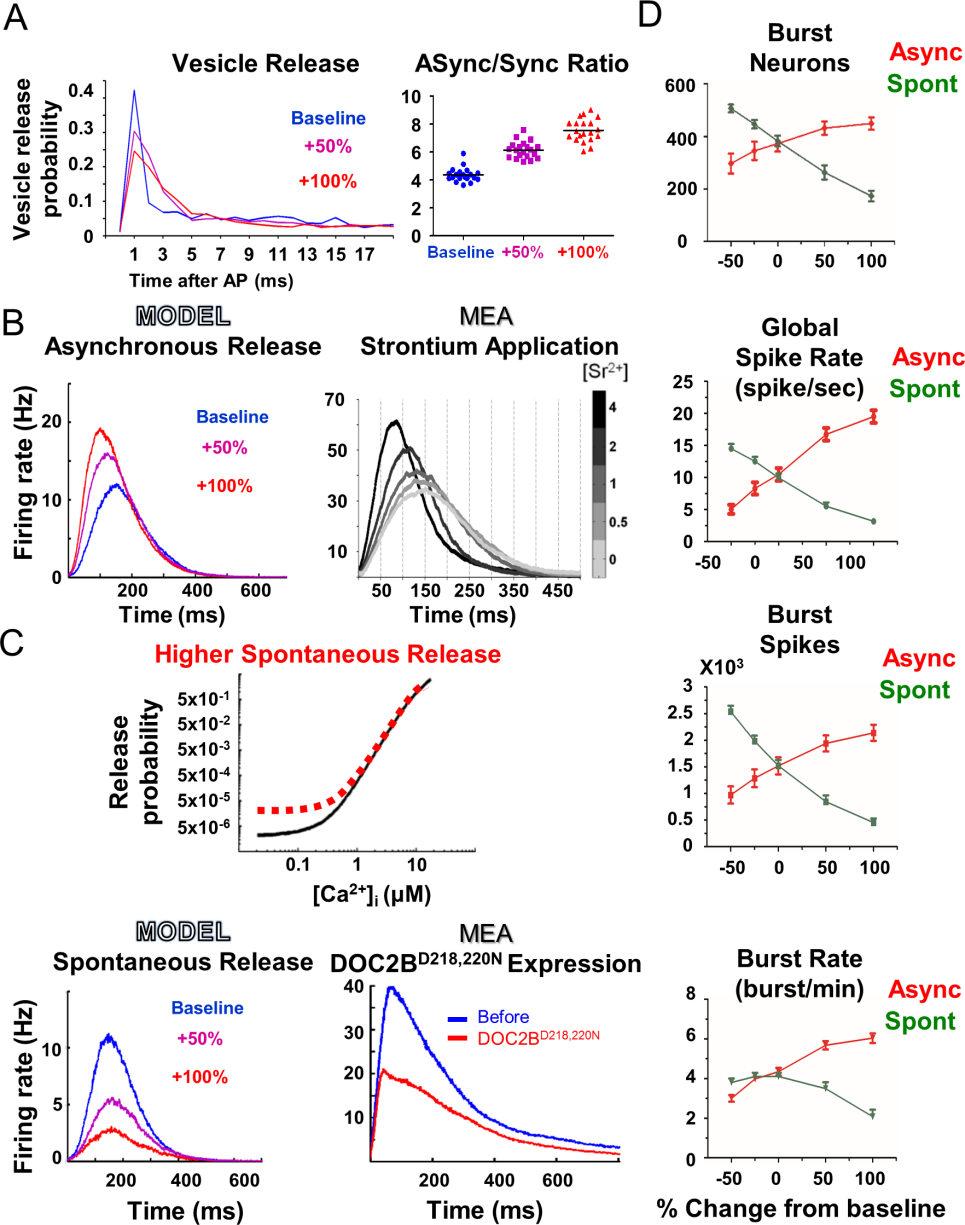
Model demonstrates how an increase in asynchronous release, and not spontaneous release, enhances neuronal network activity. **(A)** Reduction of calcium clearance rate redistributes single-neuron vesicle release (left) and increases the proportion of asynchronous release to synchronous release in the model (right). **(B)** Higher simulated asynchronous release increases network burst firing rate (left). Experimentally, the increase in asynchronous release induced by strontium application is correlated with the increase in the network burst maximum firing rate (right; modified from Lavi et al [3]). **(C)** Illustration of the change in the release function leading to higher spontaneous release probability at low intracellular calcium concentration ([Ca^2+^]_I_; top panel). Higher simulated spontaneous release reduces network burst firing rate (bottom left), in agreement with the reduction in activity induced by the expression of DOC2B^D218,220N^ (right) in the experimental MEA recordings. **(D)** Opposite effects of asynchronous and spontaneous release on network activity. In general, higher asynchronous release increases network activity whereas higher spontaneous release reduces network activity (error bars show SEM).

We first verified that slower calcium clearance increases the ratio of asynchronous to synchronous release in the model. We followed the change in the probability for vesicle release from single neurons up to 50 ms after an AP, under different calcium-efflux rates (Fig 2A; see Methods). The ratio of asynchronous to synchronous release (Fig 2A right panel; 'ASync' and 'Sync', correspondingly) increased as calcium efflux was reduced [70]. Notably, the increase in asynchronous release did not increase the total neuronal output of a single neuron but only spread the release over a longer time.

We then examined how asynchronous release affects the activity profile in the network burst (Fig 2B). Gradually increasing asynchronous release in the model enhanced the network burst firing rate (Fig 2B; +100%, left panel), similar to the experimental results of increasing strontium concentration (Fig 2B right panel). Both manipulations also decreased the time from burst onset to its peak. Interestingly, even when we increased the number of neurons in the network 10-fold (8000 instead of 800) and also when we increased the number of synapses per neuron 10-fold (10 instead of 1), enhanced asynchronous release facilitated network burst firing rate and decreased the network burst's time to peak (S5 Fig). This supports the power of the model in mimicking experimental results and suggests that asynchronous release has a profound effect on neuronal network activity.

Next, we focused our analysis on the manipulation of spontaneous release and tested its effects on the network activity. Experimentally, spontaneous release was increased by overexpressing a DOC2B mutant, DOC2B^D218,220N^, that is known to increase spontaneous release [55]. Computationally, spontaneous release was elevated by increasing the Pr at resting calcium (Fig 2C top panel; Eq 5). This manipulation increases the probability of vesicle release under resting conditions, which is the basic definition of spontaneous release [25]. The increase in spontaneous release in the model led to a significant decrease in the network burst activity, as evidenced by the reduced network burst activity profile and the lower global spike rate in each network burst (Fig 2C and 2D). This manipulation recreated the experimental data of DOC2B^D218,220N^ overexpression (Fig 2C; compare bottom left panel, model, to bottom right panel, experiment) while reducing the number of spikes and the number of neurons in the network bursts (Fig 2D). Comparison of the changes induced by both manipulations established their opposite effects on network activity (Fig 2D); while asynchronous release was positively correlated with network burst activity, spontaneous release was anticorrelated. This means that specific activity properties can change in the same direction by an increase in asynchronous release or a decrease in spontaneous release, or vice versa. These opposite effects were more prominent in the global spiking rate and network burst spikes; however, the burst rate, for example, displayed a more prominent difference between spontaneous and asynchronous release upon an increase in the corresponding parameter (Fig 2D); while higher spontaneous release reduced network burst frequency, lower spontaneous release did not change it (Fig 2D, bottom panel). Therefore, it is important to examine the combination of various network activity parameters to determine the overall effect on the network activity.

Next, we examined whether the model recreates the higher-level effects on network activity patterns observed in the experimental results [3]. Evidently, higher asynchronous release in the model significantly increased, while spontaneous release reduced the ratio of neurons participating in the network bursts (S6 Fig). This was measured by classifying network bursts into "full " or "aborted" bursts [3,44]. Moreover, analysis of the normalized network burst synchronization in the simulation showed that elevated asynchronous release also increases network burst synchronization, primarily around the peak of the network burst (S6 Fig). These analyses were in agreement with the experimental findings and showed that the model successfully recreates the response to the manipulation of asynchronous and spontaneous release. Thus, using the *in silico* model, we manipulated specific steps in the release process and linked them to specific experimental changes. Hence, the model reaffirmed a wide range of experimental analyses, from basic firing rate to high-level network synchronization parameters. The high reliability of the *in silico* model in reconstructing experimental findings allowed us to utilize it to explore the neuronal mechanisms underlying the findings and uncover the model parameters and factors that govern network activity. Specifically, the model allowed us to follow neuronal parameters, such as changes in the various vesicle pools, which are unavailable experimentally.

### Analysis of vesicular pool dynamics

**Fig 3:**
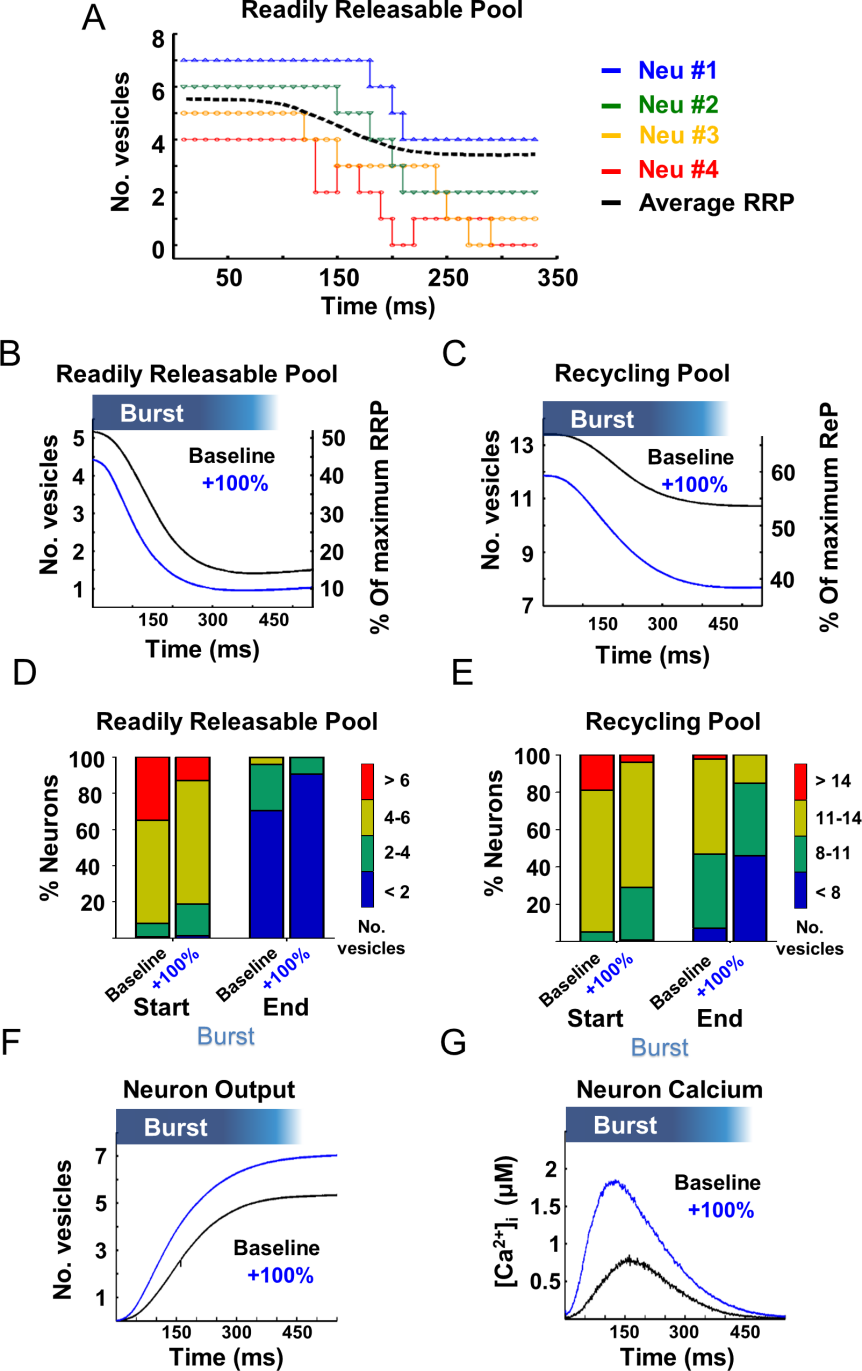
Asynchronous release utilizes the synaptic recycling pool (ReP) to elevate synaptic release during bursts. (**A**) Analysis of the number of vesicles in 4 representative neurons shows discrete changes in the number of vesicles throughout a single network burst (dotted lines). The stepwise increase in 2 of the neurons (marked in red and orange) represents the replenishment dynamics throughout the network burst, (black dashed line is the average readily releasable pool [RRP] depletion from all neurons participating in the network burst). Analysis of the average RRP depletion **(B)** and the average ReP consumption **(C)** over all neurons from all bursts in 10 simulations shows that higher asynchronous release ('+100%', blue line) leads to greater utilization of vesicles from the ReP, while the RRP is depleted by similar levels with or without changes in asynchronous release. Cumulative proportion of the average number of vesicles in the RRP **(D)** and ReP **(E)** across all neurons at the beginning (start, left) and end (end, right) of the network burst. Following an increase in asynchronous release, each neuron contributes ~2 extra vesicles within the first 300 ms of the burst **(F)**. Slower calcium efflux rate drives faster and larger accumulation of calcium during the network burst **(G)**.

We analyzed the vesicle pool dynamics and [Ca^2+^]_i_ of the model neurons under baseline release levels (Baseline) and under enhanced asynchronous release (+100%). Fig 3A demonstrates changes in the number of RRP vesicles in 4 representative neurons throughout a single network burst. Each neuron displayed different release patterns from the RRP but all displayed a certain degree of vesicle depletion (Fig 3A, 3B and 3D). Analysis of the average RRP occupancy in all neurons in all network bursts (in 10 simulations) showed that during the burst, the RRP are depleted by the same percentage under both baseline and enhanced asynchronous release conditions (Fig 3B). Further analysis of the average RRP content showed that most neurons have more than 4 vesicles in the RRP at the onset of the network burst and less than 2 vesicles at its termination (out of a maximum occupancy of 10 vesicles in the RRP; Fig 3D). It can be suggested that under these conditions, where more than 70% of the neurons have less than 2 vesicles left in the RRP (i.e. less than 20% of the entire synaptic reservoir is available), network bursts are terminated. This is not surprising but rather provides a clear connection between vesicle pool depletion and burst termination and a mechanistic explanation for previous experimental results [4,71].

This analysis could not explain the increase in network activity under enhanced asynchronous release and therefore we continued to examine the changes in ReP dynamics, which transfers vesicles to the RRP through calcium-dependent vesicle priming. The same analysis applied to the ReP showed that asynchronous release manipulation causes enhanced consumption and larger depletion of vesicles from this pool (Fig 3C); while only 7% of the neurons had less than 8 vesicles in the ReP at the time of network burst termination under baseline conditions, ~46% of the neurons had less than 8 vesicles under enhanced asynchronous release at the time of network burst termination (Fig 3E). On average, approximately 2–3 additional vesicles were consumed from the ReP during a network burst under enhanced asynchronous release (an increase of 10–15% in total synaptic release, on average; Fig 3D). This suggests that the ReP is the source for the higher output following elevated asynchronous release and that asynchronous release, driven by slower calcium clearance, relies on the replenishment rate of the ReP for support of the increased network activity.

To examine this hypothesis, we determined the average cumulative neuronal output throughout the burst (Fig 3F). On average, each neuron with a higher asynchronous release contributed ~2 more vesicles within the first 300 ms of the burst overall. This accumulated increase underlies the higher network activity and synchronization during the bursts; it also supports our hypothesis that the ReP is the source vesicle pool contributing to this network effect. The lower calcium efflux rate from the presynaptic terminal allows faster and larger accumulation of free calcium throughout the network burst (Fig 3G). This, in turn, has two important implications in the neuronal release dynamics throughout the burst: 1) higher calcium levels lead to higher Pr; 2) higher calcium levels increase the vesicle transition rate from ReP to RRP (much like the calcium-dependent vesicle replenishment hypothesis). Thus, the model revealed that the higher asynchronous release temporally increases the Pr and vesicle availability, causing enhanced neuronal network activity only during bursts. Furthermore, this analysis pointed to the ReP as the vesicle pool that supports this increase in neuronal vesicle release and network synchronization.

### Examining the predictive power of the model through manipulation of vesicle priming rate

**Fig 4:**
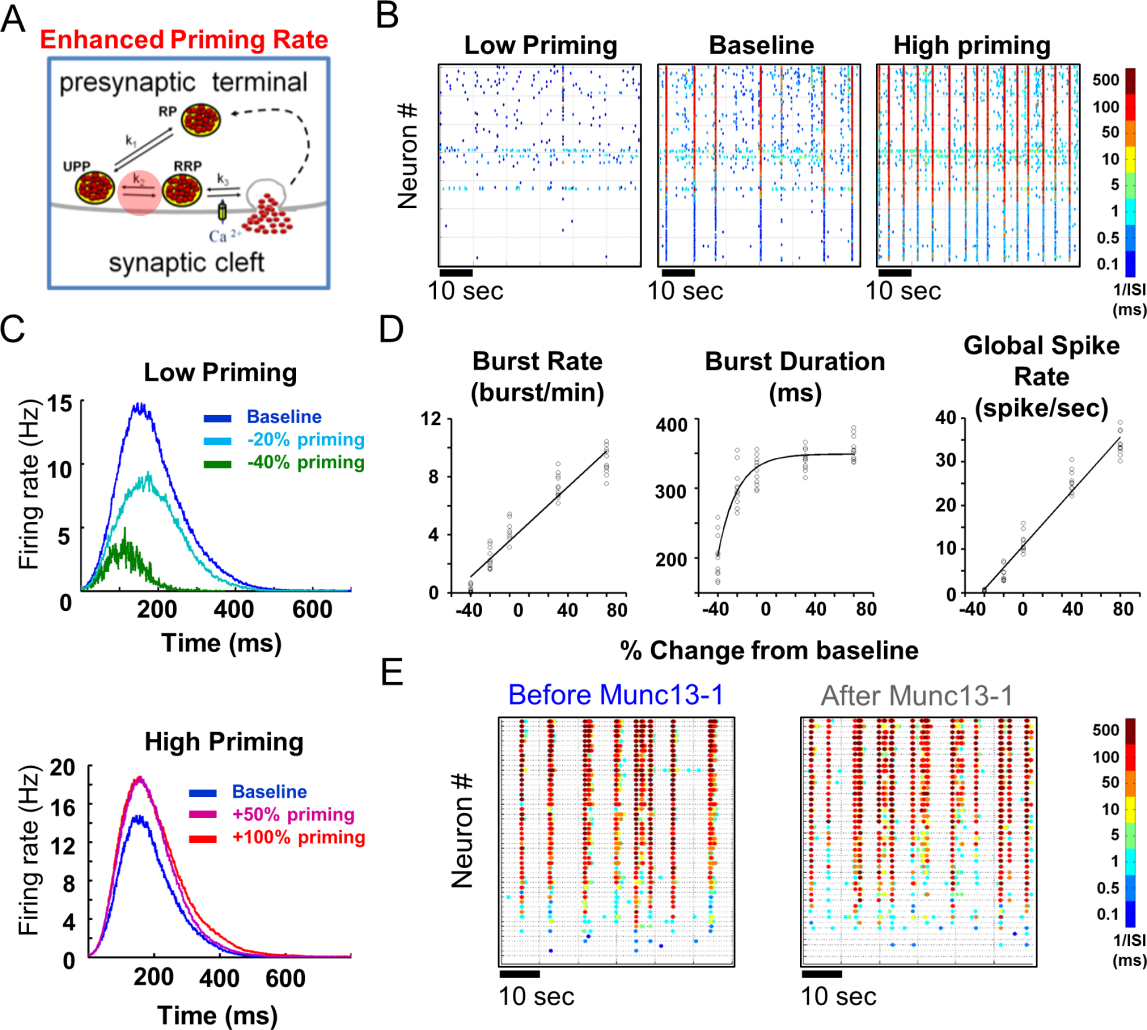
Vesicle priming rate governs the rate of network bursts. **(A)** Priming rate manipulation was simulated by changing the maximum vesicle transition rate from the recycling pool (ReP) to the readily releasable pool (RRP) (red transparent circle). **(B)** Color-coded raster plots demonstrate that lower priming rate decreases ('Low'; left panel) and higher priming rate increases ('High'; right panel) network burst frequency and overall network activity (each dot denotes a neuronal action potential, colors code for average firing rate). **(C)** Analysis of network burst profiles shows that lower priming rate reduces firing rate throughout network bursts (top panel), while higher priming rate increases firing rate throughout network bursts (bottom panel). **(D)** Analysis of network burst activity parameters shows that while higher priming rate increases the frequency of network bursts and the overall spike rate, it does not change the duration of the network bursts; this suggests the involvement of other presynaptic release processes in regulating the duration of network bursts. **(E)** Spontaneous activity recorded from a neuronal network cultured on MEA before (left) and after (right) twofold expression of Munc13-1 (each dot denotes spike from an electrode, colors code for average firing rate). In agreement with the high priming demonstrated in A, Munc13-1 expression clearly increased the frequency of network bursts. ISI, inter-spike interval.

Our model presented us with an opportunity to predict the effect of manipulations, which can be later examined experimentally. We were therefore interested in testing how changes in priming rate at the single-neuron level affect network activity. To implement this manipulation, we changed the maximum rate of vesicle transition from ReP to RRP (Fig 4A, circled red marker; parameter 'Rmax' in Fig 1E; Eq 7 *τ*_*ReP*→*RRP*_). A comparison of raster plots showed that as the priming rate increases, the activity and frequency of the bursts are enhanced, while decreasing the priming rate reduced network activity (Fig 4B). Burst profile and activity parameter analyses supported these findings, suggesting that a 50% increase in priming rate would lead to ~30% increase in the maximum firing rate within the network burst (Fig 4C and 4D) and an increase of 4 bursts per minute in network burst frequency, i.e. the network displays a higher rate of oscillations following this manipulation without elevating the inter-burst activity.

Next, we overexpressed Munc13-1, a positive regulator of vesicle priming rate, in neuronal networks plated on MEA, and found that a 2-fold increase in Munc13-1 expression levels increases the frequency of network bursts by 60% (previously published in Lavi et al. [3]; Fig 4E). These results match the model predictions (Fig 4B, High priming) and suggest that changes in vesicle priming rate at the neuronal level tune the burst frequency at the network level.

## Discussion

We created a novel neuronal network computational model to investigate how changes in synaptic transmission translate to changes in neuronal network activity and affect the network oscillation and synchronization patterns. The model was based on modified LIF neurons spread on a 2D MEA-like surface, each neuron having synapses that release vesicles following a multistep process of calcium-dependent vesicle maturation and fusion. The model reliably recreated the spontaneous activity of *in vitro* neuronal networks cultured on MEA. Consequently, the model provided mechanistic explanations and predictions for experimental pharmacological and genetic manipulations, and most importantly, linked cellular properties of synaptic release to modulations in the oscillatory activity of neuronal networks.

### Combined calcium-driven and vesicular pool computational model recreates neuronal network activity

Current computational network models do not simulate synaptic vesicle pools or calcium-dependent processes. Many computational network models based on LIF neurons simulate neuronal activity as the sum of voltage or current input on the PM to recreate neuronal network activity. Although this voltage accumulation causes the generation of an AP in the soma, it is the calcium influx through voltage-dependent calcium channels in the presynaptic terminal that drives the actual vesicle fusion and subsequent synaptic release [72,73]. Therefore, free intracellular calcium dynamics gates the transfer of synaptic information from one neuron to the next, and the combination of calcium dynamics and vesicle release probability underlies short-term plasticity in the presynaptic terminal, a key mode of operation in several central synapses [41,74–78]. Therefore, it is highly important to integrate calcium-dependent synaptic release, as we did in the current model, into LIF neuronal models.

Although the well-established tri-state model [33] did incorporate synaptic transmission into LIF neurons, that model and its succeeding extensions [29,30,34,36–38] did not simulate synaptic vesicle pools, calcium-dependent vesicle priming or vesicle release. The evoked activity simulated in those models generated very short network oscillations (several milliseconds), significantly shorter than the network bursts observed *in vitro* in dissociated neuronal cultures by single-neuron current-clamp recordings and neuronal network MEA recordings (typically hundreds of milliseconds). Furthermore, to maintain spontaneous network activity, current or voltage are artificially injected from an external source [48–50]. The lack of biological mechanisms in the neuronal model makes it harder to infer physiologically relevant consequences and predictions.

The uniqueness of our model lies in its direct simulation of key components of the presynaptic release process, thereby revealing how changes in the release process, such as changes in release probability (Eq 5), vesicle pool size (Eqs 7 and 8), and calcium dependency (Eqs 4–7), affect neuronal network activity. This direct simulation allows us to model synchronous, asynchronous and spontaneous release as derivatives of the same calcium-dependent release mechanism with different ranges of [Ca^2+^]_i_ [25,26,41]. The model also incorporates calcium-dependent vesicle priming (Eq 6), which is usually not modeled in neuronal network models. Since our model directly simulates the presynaptic vesicle pools and calcium-dependent priming based on measured rate constants, the derivation of putative physiologically relevant mechanistic explanations from its predictions is more intuitive.

The model generates network bursts that are similar to those observed in MEA recordings (S3 Fig) in duration and firing rate, thereby enabling an investigation of mechanisms for burst termination under spontaneous neuronal activity, and linking them to the dynamics of vesicle pool depletion [79]. Note that we are not claiming that it is impossible to create network bursts without incorporation of the presynaptic release mechanism, but rather that through the structure of our model, we were able to relate the network bursts to their underlying realistic and biologically plausible presynaptic mechanisms.

Our simulation allowed us to perform long-term *in silico* experiments (which we limited to several hours), while the model clearly exhibited stability and robustness to changes in the primary gain parameters that control network activity—i.e., EPSP and connectivity ratio. In agreement with our experimental results, most of the manipulations performed in the model did not abolish the basic network bursting activity but rather manipulated the inter-burst and intra-burst spiking distributions. The fact that the basic bursting activity of the model was not diminished after these manipulations establishes the model's robustness and its provision of a stable platform to uncover the role of asynchronous and spontaneous release in neuronal network oscillatory activity.

### Asynchronous release utilizes ReP vesicles to increase neuronal output and synchronize network bursting activity

A recent intriguing experimental finding demonstrated that asynchronous release, but not spontaneous release, enhances network activity and network burst synchronization [3]. The model allowed us to test how changes in asynchronous release and spontaneous release affect network activity at the neuronal level. Supported by experimental results, the model showed that higher spontaneous release leads to lower firing rate, lower neuronal participation in network bursts and lower frequency of bursts. Higher spontaneous release reduced synchronization of the network activity by the superfluous release of vesicles throughout; this excess activity reduced the availability of releasable vesicles from the RRP during network bursts, which resulted in lower intra-network burst activity and synchronization.

The model also allowed testing whether the anticipated change following strontium application—enhanced asynchronous release—is translated into enhanced activity during the bursts, and investigating the vesicular source for this effect. The model-simulated increase in asynchronous release elevated the overall activity of the network and various network burst parameters, including synchronization and neuron participation. This was in agreement with the experimental results obtained following gradual application of strontium. These results are supported by previous evidence regarding the link between asynchronous release and reverberatory activity [54].

Previous studies have shown that rapid recovery of the RRP supports asynchronous release at the neuronal level [71], and have suggested that network bursting activity depends on the vesicle depletion rate from the RRP [4,80]. Here we suggest that during ongoing network activity, the neurons in the network are not fully depleted at the termination of the network burst. Rather, examination of the RRP and ReP of all neurons in the network showed that under baseline conditions, it is sufficient that 70% of the neurons have less than 2 vesicles in the RRP (that is, less than 20% of the overall vesicles available for immediate release in the RRP) to terminate the network burst. Analysis of the average depletion rate of the ReP and RRP (over all bursts and all neurons in 10 simulation sets) following higher asynchronous release in the model suggested that the main resources for the enhanced network activity come from the ReP. This was concluded from the fact that higher asynchronous release did not increase the total number of vesicles released from the RRP but it did increase consumption of the ReP. Interestingly, we found excellent agreement between the degree of consumption of the ReP and enhancement of release at the neuronal level: asynchronous release enhanced depletion from the ReP by ~2 vesicles and respectively, this higher asynchronous release increased the total amount of vesicles released from each neuron by approximately 2 extra vesicles, on average. These 2 extra vesicles, on average, per neuron (representing 10% of the maximum ReP occupancy) accumulated and induced large-scale enhancement of neuronal firing during the bursts. This means that as previously suggested [9], a relatively small change in presynaptic release results in profound changes in the network activity.

As synaptic release in the model depends on the intracellular level of free calcium, we followed the change in [Ca^2+^]_i_ during the network bursts; we found that following the asynchronous release manipulation, the calcium concentration reaches higher levels throughout the bursts. These calcium levels increased the effective Pr and vesicle replenishment rate in each neuron in the network (due to calcium-dependent vesicle priming). These finding are in agreement with previous studies showing that sustained synaptic release requires the contribution of vesicles from the ReP [42]. Together, these analyses explain how additional vesicles are quickly primed from the ReP into the RRP (due to faster priming rate) and are readily released (due to higher Pr), leading to faster vesicle replenishment and an overall higher neuronal output only during the burst, when calcium levels are high.

What might the effects of higher asynchronous release be on neuronal microcircuits in the brain? Measurements from rat cortical acute slices and from human cortical slices have shown that cortical fast-spiking inhibitory neurons exhibit asynchronous release as part of their spontaneous activity. Furthermore, the involvement of excess asynchronous release in inhibitory fast-spiking neurons has been linked to epileptic activity in human patients with intractable epilepsy and in the rat pilocarpine model of status epilepsy [7]. This shows that asynchronous release is a fundamental property of synaptic transmission in the brain and not merely induced by drug application [7]. Recent evidence from rats suggests that the excitation-to-inhibition ratio in the adult brain is regulated by reduced GABAergic asynchronous release, which is supported by the more efficient clearance of residual calcium [6]. This might cause alterations in brain network activity in a mechanism that we simulated for *in vitro* networks. This evidence joins a previous computational model which showed that the higher levels of asynchronous GABAergic release in the cortex of juvenile animals are counterbalanced by postsynaptic shunting inhibition to regulate synaptic transmission in the developing brain [29]. Interestingly, lack of synchronous release but enhanced asynchronous release following synaptotagmin-1 knockout in the hippocampal CA1 region did not impede acquisition of contextual fear memories, but did impair their precision. This suggests that the hippocampal CA1 region can rely on spike bursts to transfer information downstream [58]. These and other recent studies [3,5,8,81–83] demonstrate the importance of understanding in detail how changes in various types of synaptic release at the single-neuron level regulate the activity of the neuronal network in brain function and dysfunction, and further stress the importance of integrating the presynaptic release mechanism into neuronal network computational models.

### Vesicle priming rate at the neuronal level tunes network burst frequency at the network level

As suggested above, asynchronous release utilizes ReP vesicles to increase network activity. This indicates the important role of vesicle priming rate, as this process regulates the rate of vesicle transfer from the ReP to the RRP. Since the model can be utilized to recreate experimental data and examine parameters that are inaccessible experimentally, we manipulated the maximum vesicle priming rate (‘Rmax’ in Eq 7).

Manipulation of vesicle priming revealed a positive correlation between priming rate at the neuronal level and burst frequency at the network level. This model prediction was supported by the viral overexpression of Munc13-1, a presynaptic protein that positively regulates vesicle priming, in neuronal networks cultured on MEA [3]. Munc13-1's higher expression levels—twofold higher than baseline—are physiologically plausible, suggesting that tuning the priming rate might have a great impact on the activity of neuronal networks in general. To infer a direct connection between vesicle priming rate and network burst frequency, an additional experimental manipulation is required that will specifically and acutely reduce the vesicle priming rate; however, the present analysis already demonstrates the model's power in predicting how changes at the neuronal level are transformed to changes at the network level, and suggests manipulation of the frequency of network bursts by changes in the presynaptic release process. The implications of these manipulations for spontaneous neural activity in the neocortex of the behaving animal remain to be tested, together with their implications for learning and memory, as well as pathological disorders.

### Shaping network oscillations by presynaptic release mechanisms

Our model shows how *in vitro* network oscillations, in the form of network bursts, can be generated and maintained based on calcium-dependent presynaptic release mechanisms, without external stimulation or injection of current. Furthermore, the model recreates some of the complex experimental data obtained from MEA recordings. A growing body of literature is connecting network bursts *in vitro* to the "up" and "down" states displayed by neocortex brain oscillations *in vivo* [84–88]. Modulation of the oscillation between "up" and "down" states during spontaneous activity *in vivo* has been observed during slow-wave sleep, selective attention and short-term memory tasks [89–92]. Therefore, elucidating the principles of spontaneous network activity and its manipulation in culture might contribute to understanding high-order functions in the behaving animal [5,27,88,93]. Interestingly, the oscillatory nature of "up" and "down" states can be explained by a modulation of presynaptic release, and it has been suggested that while non-synchronous synaptic release might maintain the "up" state [92,94], synaptic depression can be used to terminate it and return the activity to the "down" state [95]. Thus, although our model is based on *in vitro* experimental data, it opens new avenues to examining how presynaptic release mechanisms modulate microcircuit oscillations and subsequently affect higher neural functions such as slow-wave sleep, learning and attention, or are involved in pathologic neurological disorders.

## Methods

### Setting up the model parameters

#### Network structure

The entire model was simulated in MATLAB (The Mathworks, Inc.). The code is available in the supplementary material (S1 Software). The network contained 800 modified LIF neurons located on a virtual 2D surface (30% of them were inhibitory neurons). To achieve the small-world scale-free network topology, each neuron was assigned a number of connections (degree) from a power-law distribution (generalized Pareto distribution) and connected to its nearest neighbors with a binomial probability density function based on the neurons' pair-wise Euclidean distance; this created a preferential local connection with low probability for long-distance connections. We used a connectivity ratio of 5% (average ~45 connections per neuron). Key parameters of the model were based on the literature and previously published papers [15,25,67,96–101]. The simulation stored all AP times.

#### Neuronal model

Each neuron in the model had a LIF membrane potential which evolved according to the equation:

$$\tau_{m}\frac{dV}{dt}=-V+V_{syn}$$ (1)

where τ_m_ denotes the membrane voltage decay rate of a neuron, V is the neuronal membrane potential and V_syn_ represents the synaptic input a neuron receives from other neurons.

The membrane potential V was calculated relative to a given neuron's resting level. The synaptic input voltage was modeled as the sum of postsynaptic voltages from all other neurons which have a connection to the given neuron i:

$$V_{syn}\left(i\right)=\underset{j}{\sum }A_{ij}\cdot x_{ij}\left(t\right)\cdot q$$ (2)

Based on previous studies [36], we used A_ij_ to describe the synaptic strength between neuron j (presynaptic neuron) and neuron i (postsynaptic neuron). The sign of A_ij_ represents the type of synapse between the connected neurons: when A_ij_ > 0, the neurons are connected via an excitatory synapse, when A_ij_ < 0, the neurons are connected via an inhibitory synapse. The number of vesicles released at time t from the presynaptic neuron j to the postsynaptic neuron i was represented by x_ij_(t), and the contribution of each EPSP was denoted by q and was equal to 3.16 mV: as we used a Pr of ~0.15 [101,102] and the number of vesicles in the RRP was estimated to be ~10 [97], we estimated that ~1.5 vesicles are released per AP per synapse. We simulated up to 10 synapses per neuron, meaning that 15 vesicles contributed to a single EPSP. Based on experimental measurement from pairs of neurons, we used an average EPSP voltage of 4.74 mV [49,98,103] and therefore each vesicle contributed ~0.31 mV (4.7/15 = 0.316 mV). For simplicity, we modeled one synapse for each neuron and thus we pooled all 10 synapses into 1 synapse and used the value of 3.16 mV for each EPSP. Based on previous studies, the synaptic weight was taken from the log-normal distribution [96]:

$$A_{ij}∼lnN\;\left(\mu^{Aij},\sigma^{Aij}\right),\;A_{ij}<A_{max}$$ (3)

where μ^Aij^ = -0.7835, σ^Aij^ = 1.0264 and A_max_ = 10.

At each simulation step (1 ms), the synaptic output of each neuron was determined by vesicle availability and effective Pr (which was determined from the calcium-dependent release curve [26] and adjusted for cortical neurons, as described below). Whenever the depolarization hit a fixed threshold θ (i.e. Vi(t) ≥ θ), the neuron emitted a spike and became refractory for a period τ_arp_, and its voltage was reset to a hyperpolarized voltage V_hyp_ (see parameters in Table 1).

#### Intracellular calcium pools

The free [Ca^2+^]_i_ for each neuron ([Ca^2+^]_tot_) was represented by the sum of two calcium pools to simulate bi-exponential calcium efflux [53]. It is important to note that we only simulated the concentration of free calcium, i.e. the calcium that is directly available for synaptic release, and we did not account for changes in somatic calcium concentration.

$$\begin{array}{c} {\left[Ca^{2+}\right]}_{tot}={\left[Ca^{2+}\right]}_{fast}+{\left[Ca^{2+}\right]}_{slow}+{\left[Ca^{2+}\right]}_{rest};\;{\left[Ca^{2+}\right]}_{rest}=50nM\\ \frac{d{\left[Ca^{2+}\right]}_{slow}}{dt}=-\frac{{\left[Ca^{2+}\right]}_{slow}}{\tau_{Ca_{slow}}}+CA_{SLOW}\cdot \delta \left(t-\;t_{AP}\right)\\ \frac{d{\left[Ca^{2+}\right]}_{fast}}{dt}=-\frac{{\left[Ca^{2+}\right]}_{fast}}{\tau_{Ca_{fast}}}+\left(CA_{FAST}-\;{\left[Ca^{2+}\right]}_{fast}\right)\cdot \delta \left(t-\;t_{AP}\right) \end{array}$$ (4)

The slow calcium concentration was calculated relative to a basal calcium level for a given neuron. The fast calcium pool rose to 13.6 μM (*CA*_*FAST*_) and decayed very quickly, and the slow calcium pool rose to 1.36 μM (*CA*_*SLOW*_) and decayed more slowly (*τ*_*Ca*_*fast*__ = 1 *ms*, *τ*_*Ca*_*slow*__ = 31 *ms*). The calcium level could drop to a minimum baseline calcium level, [Ca^2+^]_rest_, set at 50 nM. As previously suggested [25], the ratio of calcium concentrations between the pools was approximately 1 to 10. Manipulations of calcium clearance in the paper were performed by changing both the fast and slow time constants by the same ratio. Each AP induced a stepwise increase of 500 nM calcium in the neuron (accumulated in the slow calcium pool), consequently increasing the release probability.

#### Calcium-dependent release probability

The calcium-dependent release probability function was based on the enclosed sigmoid function that depends on the total [Ca^2+^]_i_ (parameters detailed in Table 1):

$$\text{Pr}\left({\left[Ca^{2+}\right]}_{tot}\right)=\frac{\alpha }{1+e^{-\beta \cdot {\text{log}}_{10}{\left[Ca^{2+}\right]}_{tot}+\gamma }}+\delta$$ (5)

Note: parameter 'α' in this expression corresponds to 'calcium_release_A' in Table 1.

This Calyx of Held calcium-dependent release-rate curve [26] was fitted with a sigmoid function [104] and the maximum Pr was reduced to 0.15 to fit the lower Pr of cortical synapses [101,102].

#### Vesicle pool dynamics

The probability of vesicle release was determined at each simulation step from binomial trials for each neuron. Vesicles could only be released from the RRP by the following equation:

$$X_{i}:X_{i}∼B\left(\lfloor {RRP}_{i}\rfloor ,\text{Pr}\left({\left[Ca^{2+}\right]}_{{tot}_{i}}\right)\right)$$ (6)

where X_i_ comes from a binomial distribution (denoted B), RRP_i_ is the number of vesicles in the RRP of neuron i and [*Ca* ^2+^]_*tot*_*i*__ is the total free calcium concentration of neuron i. At each step, the RRP was replenished from the ReP by the calcium-dependent vesicle priming process described by:

dRRPidt=RePiτReP→RRP([Ca2+]toti)−RRPiτRRP→ReP−XiτReP→RRP([Ca2+]toti)=1Rmax⋅[Ca2+]toti[Ca2+]toti+KdτRRP→ReP=τReP→RRP([Ca2+]toti)⋅RRPfullRePfull (7)

The amount of vesicles transferring between the ReP and the RRP was based on the Michaelis–Menten function [15,67] and was determined by the overall calcium level for each neuron. Accordingly, the RP was replenished at a constant rate and replenishment of the ReP from the RP and RRP was calculated according to predefined rate constants (Table 1):

$$\begin{array}{c} \frac{dReP_{i}}{dt}=\frac{RRP_{i}}{\tau_{RRP\to ReP}}-\frac{ReP_{i}}{\tau_{ReP\to RRP}\left({\left[Ca^{2+}\right]}_{tot_{i}}\right)}+\frac{RP_{i}}{\tau_{RP\to ReP}}-\frac{ReP_{i}}{\tau_{ReP\to RP}}\\ \frac{dRP_{i}}{dt}=\frac{RP_{full}-RP_{i}}{\tau_{\to RP}}+\frac{ReP_{i}}{\tau_{ReP\to RP}}-\frac{RP_{i}}{\tau_{RP\to ReP}}\\ \tau_{ReP\to RP}=\tau_{RP\to ReP}\cdot \frac{ReP_{full}}{RP_{full}} \end{array}$$ (8)

where ReP_i_ and RP_i_ denote the number of vesicles in the ReP and RP of neuron *i*, respectively. Upon vesicle release, the voltage of the postsynaptic neuron changed according to the EPSP voltage (and the number of fused vesicles), the type of synapse (inhibitory neurons contribute negative voltage) and the synaptic weight.

### Analyses and visualization

#### Data analysis and visualization

Color-coded raster plots were generated by calculating the average firing frequency for each spike (calculated from the average interval of the spike from the following and preceding ones). Analyses of network burst activity, such as burst detection, burst statistics, burst profile analysis, burst synchronization and "full"/"aborted" burst classification were based on a previous study [3]. Briefly, potential network burst peaks were identified when the firing rate of the active neurons in the network crossed a predefined threshold (usually 5% of the maximum firing rate; active neurons display an average firing rate of >0.02 s^-1^). Next, burst initiation and termination times were identified by defining the maximum allowed inter-spike interval in the burst (usually 100 ms). The time of the first and last spikes in the burst defined the burst initiation and terminations times, respectively.

#### Network connectivity analysis

Shortest path analysis was based on the Floyd–Warshall algorithm [105,106]. Clustering coefficient analysis was based on the ratio between the number of triangles and the number of paths of length 2 in the network [107]. The normalized clustering coefficient was the ratio between the clustering coefficient in the chosen network configuration and the clustering coefficient in an equivalent Erdös–Rényi (E–R) random network with the same number of neurons and connections. Analysis of small-world index was the ratio between the normalized clustering coefficient and the normalized shortest path [107]; a small-world index > 1 indicated a small-world connectivity in the network; higher values denoted a higher degree of small-worldness [107]. Analysis of the connectivity parameters can be found in the supplementary material.

#### Single-neuron parameter analysis

Single neuron parameters, such as vesicle pool dynamics and [Ca^2+^]_i_ ([*Ca* ^2+^]_*tot*_*i*__), were analyzed for all neurons in a subset of simulations (10 simulations). The analysis focused on the network burst periods by averaging the pool size of all neurons for each simulation step. Next, all network bursts were aligned by their initiation time (the time of the first spike in the burst) and the average pool size throughout the burst was calculated. Analysis of [*Ca* ^2+^]_*tot*_*i*__ was performed in the same manner.

#### Burst classification and synchronization analysis

Classification of bursts into "full and "aborted" was based on the number of neurons participating in the network burst [44]. Bursts in which more than 50% of the electrodes participated were defined as "full" bursts, and other bursts were defined as "aborted". Network burst synchronization analysis [3] was calculated from the average pairwise Pearson correlation for all active electrodes or neurons that were active in the network bursts throughout the experimental recording or the model simulation, respectively. To account for inter-culture variability, synchronization was standardized by firing rate and normalized to baseline conditions; ‘baseline’ was the condition before the genetic or pharmacological manipulation in the experimental data and before the change in the parameters’ values in the model.

#### Primary gain parameters chosen for robustness analysis

We chose two important gain parameters to examine the stability and robustness of the model. EPSP was chosen as a primary gain parameter because the neuron model was based on a LIF mechanism and could only generate AP upon summation of the inputs arriving in a limited time window. This is very similar to accumulation of EPSP voltage during spatial or temporal summation. This means that if a neuron receives low voltage at each input (quantal voltage) or receives a low frequency of inputs, it will not integrate the inputs into APs, which in turn will not support the propagation of synaptic transmission throughout the network. The connectivity of the network was chosen as a primary gain parameter because a low connectivity ratio translates to fewer connections for each neuron, and each neuron subsequently receives a lower frequency of synaptic input. It is also important to note that excess synaptic input (by either very high frequency or very high voltage) can also suppress the network bursting activity, as it might lead neurons to spike constantly and deplete synaptic resources.

#### Neuronal culturing on MEA

All experimental procedures were approved by the Chancellor’s Animal Research Committee at Tel Aviv University (approval #L12-066), in accordance with the regulations and guidelines. Cortical neuronal network preparation and MEA plating were perform as described in a previous work [3]. Briefly, cortical neuronal cultures were prepared from newborn P1 mice. Cortical tissue was separated from the hippocampus and was then mechanically dissociated and the cells were plated in Neurobasal-A supplemented with B-27, GlutaMAX-I, antibiotics (penicillin–streptomycin; Invitrogen, Carlsbad, CA, USA) and 5% fetal calf serum to support glial cell growth on the day of culture preparation. On the following day and twice a week thereafter, the medium was exchanged with growth medium, which was essentially the plating medium without the serum. MEA plates were cultured with one million cells in a 100-μl drop applied to the middle of the plate (final cell density was estimated at 2500 to 3000 cell/mm^2^). Cultures were kept in an atmosphere of 5% CO_2_ and 95% air at 37°C and were recorded 2 weeks after plating (no significant difference in culture age), before and after overexpression of the following proteins: DOC2B separated by an internal ribosome entry sequence (IRES) from GFP (DOC2B; 15 cultures), DOC2B^D218,220N^ separated by an IRES from GFP (DOC2B^D218,220N^; 9 cultures) and Munc13-1 conjugated to GFP (Munc13-1; 10 cultures). Strontium experiments were performed on naive cultures to which 2 mM strontium (Sigma) and 4 mM EGTA (Sigma) were applied to replace the existing Ca^2+^ ions in the external solution as previously described [108]. To enable long-term (hours) recordings before and after virus application, custom-made incubation chambers consisting of a glass cylinder and a plastic ring were glued to MEA plates with Sylgard 184 (Dow Corning, Midland, MI, USA). The MEA plates used in this study consisted of 60 Ti/Au/TiN+iR electrodes of 30 μm diameter and 500 μm spacing and were pretreated with polyethyleneimine (1:5000, Sigma-Aldrich) to promote neuron adhesion.

## Supporting Information

S1 Fig DOC2B expression increases the firing rate and number of neurons participating in network bursts. **(A)** Color-coded raster plots displaying 1 min of spontaneous activity before (left) and 6 h after (right) overexpression of DOC2B. For every electrode (in each row), each spike is colored by the average inter-spike interval (1/ISI; electrodes are ordered by activity level, most active electrodes at the top). **(B)** Following DOC2B overexpression, the spiking frequency recorded by the electrodes within the network burst increases and more electrodes participate in the network bursts (2 s of spontaneous activity enlarged from the respective plot in A marked by black arrow; modified from Lavi et al. [3]).
(DOCX)

S2 Fig Network connectivity analysis confirms small-world scale-free connectivity. **(A)** Quantitative analysis of the network connectivity was utilized to examine how the chosen connectivity balances between small-world (left panel) and scale-free (right panel) connectivity properties. **(B)** As expected, the increase in connectivity ratio is perfectly correlated with the increase in the average connectivity degree, i.e. the average number of connections each neuron creates (top left panel; R = 1, Pearson correlation). Normalized clustering coefficient analysis (bottom right panel) shows that the baseline connectivity (5%) has a significantly higher clustering coefficient compared to a random network, indicating that the network topology answers the basic requirements for small-world and scale-free networks [109–111]. Small-world index [107] quantitatively measures the small-worldness of the network topology (top right panel). Under baseline conditions, the network topology is between small-world and scale-free topology (small-world index > 1). Average shortest path analysis (bottom left panel) supports this analysis by indicating that the average shortest path was longer than the path expected from a scale-free connectivity but shorter than the path expected from a small-world connectivity.
(DOCX)

S3 Fig Calcium-dependent neuronal release mechanisms generate spontaneous network activity, which is more similar to experimental data. **(A)** Raster plot displaying simulated spontaneous network activity (top panel) maintained by neuronal calcium-dependent release mechanisms (lower panel displays a representative network burst marked by arrow in the upper panel). (**B**) Raster plot displaying simulated spontaneous network activity maintained by current injection to neurons but without calcium-dependent release mechanisms (lower panel displays a representative network burst marked by arrow in the upper panel). (**C**) Comparison of *in silico* network activity parameters to experimental data shows that the neuronal models based on calcium-dependent release (‘Ca-dependent release’) generate network bursts which are more similar to network bursts recorded from neuronal network cultured on microelectrode arrays (‘MEA’) in comparison to neuronal models which receive only current injection. Time-to-peak, time from burst initiation to peak firing rate; Neuron participation, percentage of neurons which are active in network bursts. *P < 0.05, ***P < 0.001, one-way ANOVA; error bars show SEM.
(DOCX)

S4 Fig Simulated neuronal network activity is stable under manipulation of EPSP and connectivity ratio. **(A)** Raster plot of a typical simulation run of neuronal network activity: each 30-min period simulates the neuronal network activity under different conditions. Percentage denotes change from baseline EPSP. **(B)** Increase in EPSP is significantly and positively correlated with overall firing rate in the network (spikes/sec), the number of spikes in each burst (Burst spikes), the frequency of network bursts (Burst/min) and the duration of the network bursts (*P* < 0.001 under regression analysis). The activity of the simulated neuronal network is also stable under manipulation of its connectivity ratio (the percentage of actual connections in the network out of all possible connections in the network). **(C)** Raster plots of spontaneous activity of 3 networks with various connectivity ratios (2.5%, 5% and 10%; 5% is the baseline connectivity ratio used in all simulations). **(D)** Connectivity ratio is positively correlated with burst neurons, spikes, duration and frequency (*P* < 0.001 under exponential regression analysis). Note that while the EPSP changes induce linear changes, the connectivity ratio induces exponential changes in the network activity parameters.
(DOCX)

S5 Fig Primary effects of asynchronous release on network activity are maintained following substantial changes to network structure. (**A**) Raster plot of spontaneous activity of a simulated neuronal network with a 10-fold increase in the number of neurons (8000 neurons, top panel; lower panel displays a representative network burst marked by arrow in the upper panel). (**B**) Raster plot of spontaneous activity of a simulated neuronal network with a 10-fold increase in the number of synapses per neuron (10 independent synapses per neuron, top panel; lower panel displays a representative network burst marked by arrow in the upper panel). Under a 10-fold increase in the number of neurons (**C**) or 10-fold increase in the number of synapses per neuron (**D**), the enhanced asynchronous release still increases peak network burst firing rate and reduces the network burst time to peak.
(DOCX)

S6 Fig Enhanced asynchronous release increases network synchronization and percentage of "full" bursts. **(A)** Higher asynchronous release following DOC2B overexpression and strontium application significantly increases the ratio of "full" bursts, while higher spontaneous release following DOC2B^D218,220N^ decreases this ratio compared to GFP control cultures (top panel; *P < 0.05, **P < 0.01, under one-way ANOVA; error bars show SEM; modified from Lavi et al. [3]). In the model, analysis of the "full" vs. "aborted" bursts follows the experimental results (bottom panel; ***P < 0.001 under one-way ANOVA; error bars show SEM); higher spontaneous release in the model significantly decreases the ratio of "full" bursts while the increase in asynchronous release in the model increases the same ratio. **(B)** Network burst synchronization analysis shows that enhanced asynchronous release, induced by DOC2B overexpression or strontium application, significantly increases network burst synchronization, while higher spontaneous release frequency following DOC2B^D218,220N^ overexpression significantly reduces network burst synchronization (top panel; each line represents the average change from baseline conditions in pairwise Pearson correlation for all active electrodes in the network burst; 15 DOC2B recordings, 6 strontium recordings, 9 DOC2B^D218,220N^ recordings; *P < 0.05, **P < 0.01, ***P < 0.001, ANOVA for repeated measurements; error bars show SEM; modified from Lavi et al. [3]). In agreement with the experimental findings, analysis of network burst synchronization in the model shows that while spontaneous release significantly reduces network burst peak synchronization, asynchronous release significantly increases peak network burst synchronization (bottom panel; *P < 0.05, **P < 0.01, ***P < 0.001, ANOVA for repeated measurements; error bars show SEM). The increase in the model is most significant during the peaks of the bursts and seems to be shorter than the experimental effect, perhaps due to the reduced variability in the model's burst duration.
(DOCX)

S1 Software The zip archive contains the complete computational model. A readme file, included in the archive, briefly describes the general flow of the simulation. The demo scripts included in the archive reproduce the effects of asynchronous release on neuronal network activity as demonstrated in Fig 2. The code is written in Matlab and requires the Matlab programming environment (version 7.3, The Mathworks Inc.).
(ZIP)
