# Supplementary material for: Shaping Neuronal Network Activity by Presynaptic Mechanisms
Source: PLoS Comput Biol. 2015 Sep 15;11(9):e1004438. doi: 10.1371/journal.pcbi.1004438 (PMC4570815; doi:10.1371/journal.pcbi.1004438)
Supplement: S1 Fig — (A) Color-coded raster plots displaying 1 min of spontaneous activity before (left) and 6 h after (right) overexpression of DOC2B. For every electrode (in each row), each spike is colored by the average inter-spike interval (1/ISI; electrodes are ordered by activity level, most active electrodes at the top). (B) Following DOC2B overexpression, the spiking frequency recorded by the electrodes within the network burst increases and more electrodes participate in the network bursts (2 s of spontaneous activity enlarged from the respective plot in A marked by black arrow; modified from Lavi et al. [3]). (DOCX) [file pcbi.1004438.s001.docx]

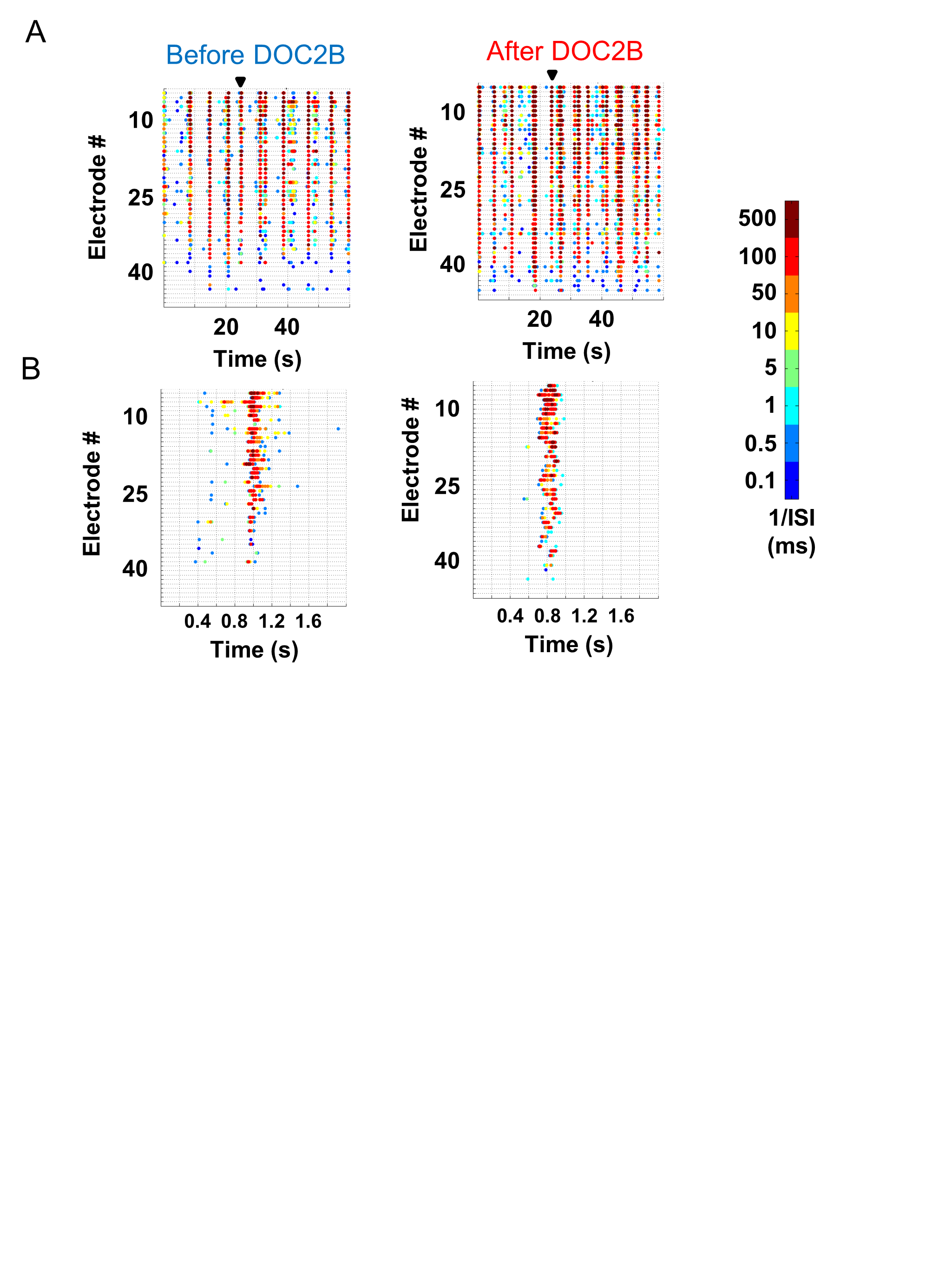


**Figure S1. DOC2B expression increases the firing rate and number of neurons participating in network bursts.** **(A)** Color-coded raster plots displaying 1 min of spontaneous activity before (left) and 6 h after (right) overexpression of DOC2B. For every electrode (in each row), each spike is colored by the average inter-spike interval (1/ISI; electrodes are ordered by activity level, most active electrodes at the top; modified from Lavi et al. [3]). **(B)** Following DOC2B overexpression, the spiking frequency recorded by the electrodes within the network burst increases and more electrodes participate in the network bursts (2 s of spontaneous activity enlarged from the respective plot in A marked by black arrow; modified from Lavi et al. [3]).
