# Supplementary material for: Shaping Neuronal Network Activity by Presynaptic Mechanisms
Source: PLoS Comput Biol. 2015 Sep 15;11(9):e1004438. doi: 10.1371/journal.pcbi.1004438 (PMC4570815; doi:10.1371/journal.pcbi.1004438)
Supplement: S2 Fig — (A) Quantitative analysis of the network connectivity was utilized to examine how the chosen connectivity balances between small-world (left panel) and scale-free (right panel) connectivity properties. (B) As expected, the increase in connectivity ratio is perfectly correlated with the increase in the average connectivity degree, i.e. the average number of connections each neuron creates (top left panel; R = 1, Pearson correlation). Normalized clustering coefficient analysis (bottom right panel) shows that the baseline connectivity (5%) has a significantly higher clustering coefficient compared to a random network, indicating that the network topology answers the basic requirements for small-world and scale-free networks [109–111]. Small-world index [107] quantitatively measures the small-worldness of the network topology (top right panel). Under baseline conditions, the network topology is between small-world and scale-free topology (small-world index > 1). Average shortest path analysis (bottom left panel) supports this analysis by indicating that the average shortest path was longer than the path expected from a scale-free connectivity but shorter than the path expected from a small-world connectivity. (DOCX) [file pcbi.1004438.s002.docx]

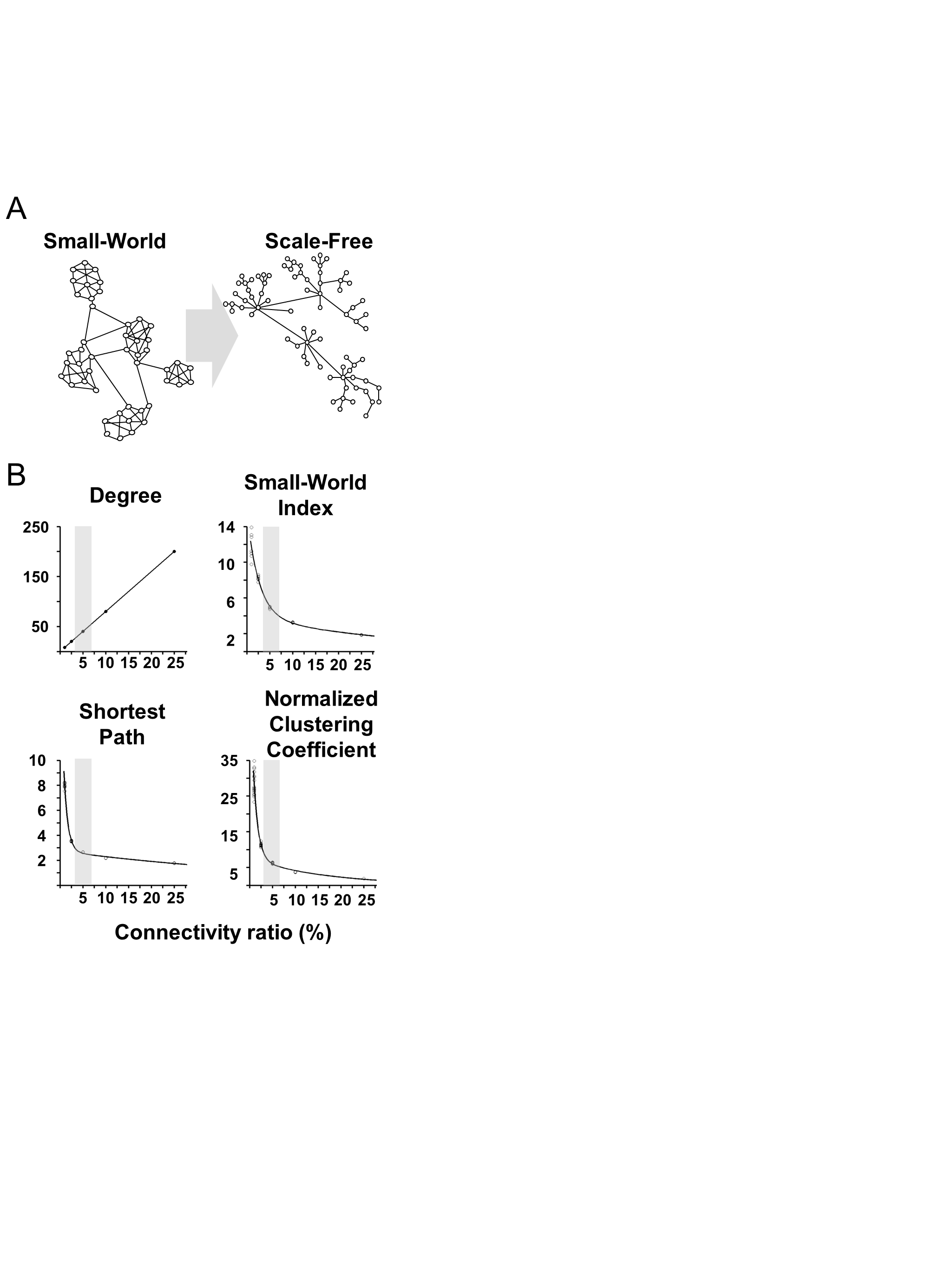


**Figure S2. Network connectivity analysis confirms small-world scale-free connectivity.** **(A)** Quantitative analysis of the network connectivity was utilized to examine how the chosen connectivity balances between small-world (left panel) and scale-free (right panel) connectivity properties. **(B)** As expected, the increase in connectivity ratio is perfectly correlated with the increase in the average connectivity degree, i.e. the average number of connections each neuron creates (top left panel; R = 1, Pearson correlation). Normalized clustering coefficient analysis (bottom right panel) shows that the baseline connectivity (5%) has a significantly higher clustering coefficient compared to a random network, indicating that the network topology answers the basic requirements for small-world and scale-free networks [109–111]. Small-world index [107] quantitatively measures the small-worldness of the network topology (top right panel). Under baseline conditions, the network topology is between small-world and scale-free topology (small-world index > 1). Average shortest path analysis (bottom left panel) supports this analysis by indicating that the average shortest path was longer than the path expected from a scale-free connectivity but shorter than the path expected from a small-world connectivity.
