# Supplementary material for: Shaping Neuronal Network Activity by Presynaptic Mechanisms
Source: PLoS Comput Biol. 2015 Sep 15;11(9):e1004438. doi: 10.1371/journal.pcbi.1004438 (PMC4570815; doi:10.1371/journal.pcbi.1004438)
Supplement: S3 Fig — (A) Raster plot displaying simulated spontaneous network activity (top panel) maintained by neuronal calcium-dependent release mechanisms (lower panel displays a representative network burst marked by arrow in the upper panel). (B) Raster plot displaying simulated spontaneous network activity maintained by current injection to neurons but without calcium-dependent release mechanisms (lower panel displays a representative network burst marked by arrow in the upper panel). (C) Comparison of in silico network activity parameters to experimental data shows that the neuronal models based on calcium-dependent release (‘Ca-dependent release’) generate network bursts which are more similar to network bursts recorded from neuronal network cultured on microelectrode arrays (‘MEA’) in comparison to neuronal models which receive only current injection. Time-to-peak, time from burst initiation to peak firing rate; Neuron participation, percentage of neurons which are active in network bursts. *P < 0.05, ***P < 0.001, one-way ANOVA; error bars show SEM. (DOCX) [file pcbi.1004438.s003.docx]

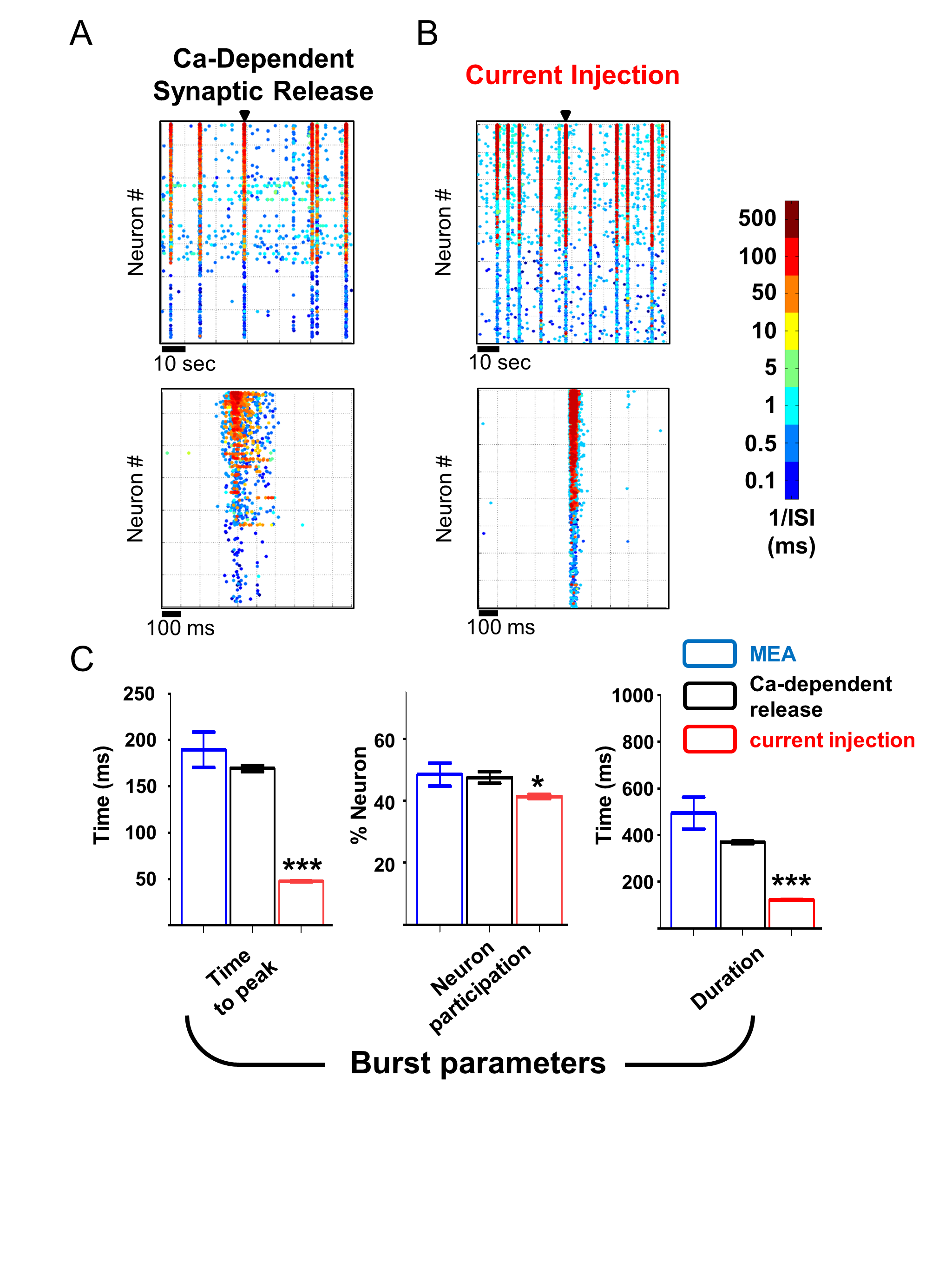


**Figure S3. Calcium-dependent neuronal release mechanisms generate spontaneous network activity, which is more similar to experimental data. (A)** Raster plot displaying simulated spontaneous network activity (top panel) maintained by neuronal calcium-dependent release mechanisms (lower panel displays a representative network burst marked by arrow in the upper panel). (**B**) Raster plot displaying simulated spontaneous network activity maintained by current injection to neurons but without calcium-dependent release mechanisms (lower panel displays a representative network burst marked by arrow in the upper panel). (**C**) Comparison of *in silico* network activity parameters to experimental data shows that the neuronal models based on calcium-dependent release (‘Ca-dependent release’) generate network bursts which are more similar to network bursts recorded from neuronal network cultured on microelectrode arrays (‘MEA’) in comparison to neuronal models which receive only current injection. Time-to-peak, time from burst initiation to peak firing rate; Neuron participation, percentage of neurons which are active in network bursts. **P* < 0.05, ****P* < 0.001, one-way ANOVA; error bars show SEM.
