# Supplementary material for: Shaping Neuronal Network Activity by Presynaptic Mechanisms
Source: PLoS Comput Biol. 2015 Sep 15;11(9):e1004438. doi: 10.1371/journal.pcbi.1004438 (PMC4570815; doi:10.1371/journal.pcbi.1004438)
Supplement: S4 Fig — (A) Raster plot of a typical simulation run of neuronal network activity: each 30-min period simulates the neuronal network activity under different conditions. Percentage denotes change from baseline EPSP. (B) Increase in EPSP is significantly and positively correlated with overall firing rate in the network (spikes/sec), the number of spikes in each burst (Burst spikes), the frequency of network bursts (Burst/min) and the duration of the network bursts (P < 0.001 under regression analysis). The activity of the simulated neuronal network is also stable under manipulation of its connectivity ratio (the percentage of actual connections in the network out of all possible connections in the network). (C) Raster plots of spontaneous activity of 3 networks with various connectivity ratios (2.5%, 5% and 10%; 5% is the baseline connectivity ratio used in all simulations). (D) Connectivity ratio is positively correlated with burst neurons, spikes, duration and frequency (P < 0.001 under exponential regression analysis). Note that while the EPSP changes induce linear changes, the connectivity ratio induces exponential changes in the network activity parameters. (DOCX) [file pcbi.1004438.s004.docx]

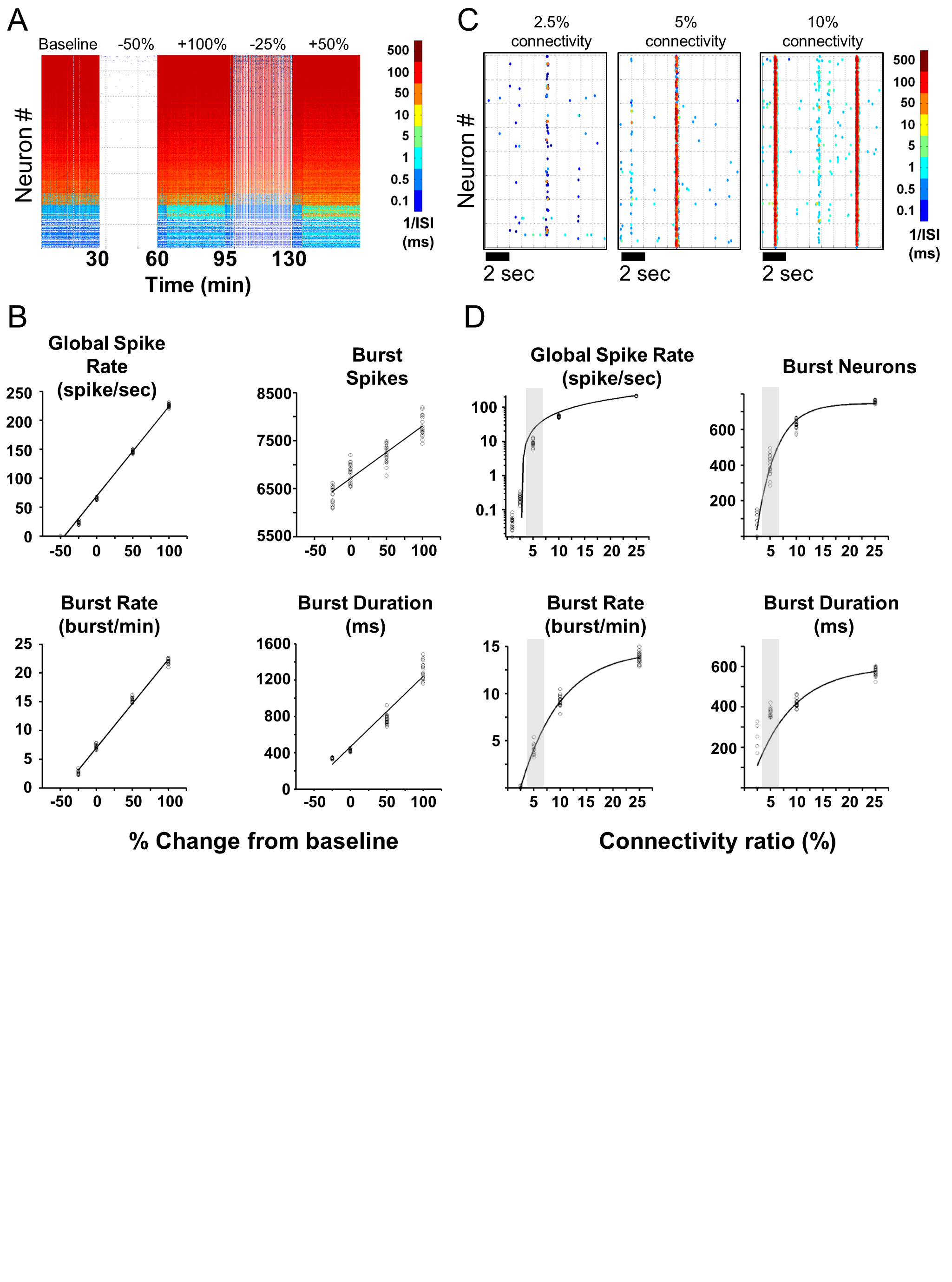


**Figure S4. Simulated neuronal network activity is stable under manipulation of EPSP and connectivity ratio. (A)** Raster plot of a typical simulation run of neuronal network activity: each 30-min period simulates the neuronal network activity under different conditions. Percentage denotes change from baseline EPSP. **(B)** Increase in EPSP is significantly and positively correlated with overall firing rate in the network (spikes/sec), the number of spikes in each burst (Burst spikes), the frequency of network bursts (Burst/min) and the duration of the network bursts (*P* < 0.001 under regression analysis). The activity of the simulated neuronal network is also stable under manipulation of its connectivity ratio (the percentage of actual connections in the network out of all possible connections in the network). **(C)** Raster plots of spontaneous activity of 3 networks with various connectivity ratios (2.5%, 5% and 10%; 5% is the baseline connectivity ratio used in all simulations). **(D)** Connectivity ratio is positively correlated with burst neurons, spikes, duration and frequency (*P* < 0.001 under exponential regression analysis). Note that while the EPSP changes induce linear changes, the connectivity ratio induces exponential changes in the network activity parameters.
