# Supplementary material for: Shaping Neuronal Network Activity by Presynaptic Mechanisms
Source: PLoS Comput Biol. 2015 Sep 15;11(9):e1004438. doi: 10.1371/journal.pcbi.1004438 (PMC4570815; doi:10.1371/journal.pcbi.1004438)
Supplement: S5 Fig — (A) Raster plot of spontaneous activity of a simulated neuronal network with a 10-fold increase in the number of neurons (8000 neurons, top panel; lower panel displays a representative network burst marked by arrow in the upper panel). (B) Raster plot of spontaneous activity of a simulated neuronal network with a 10-fold increase in the number of synapses per neuron (10 independent synapses per neuron, top panel; lower panel displays a representative network burst marked by arrow in the upper panel). Under a 10-fold increase in the number of neurons (C) or 10-fold increase in the number of synapses per neuron (D), the enhanced asynchronous release still increases peak network burst firing rate and reduces the network burst time to peak. (DOCX) [file pcbi.1004438.s005.docx]

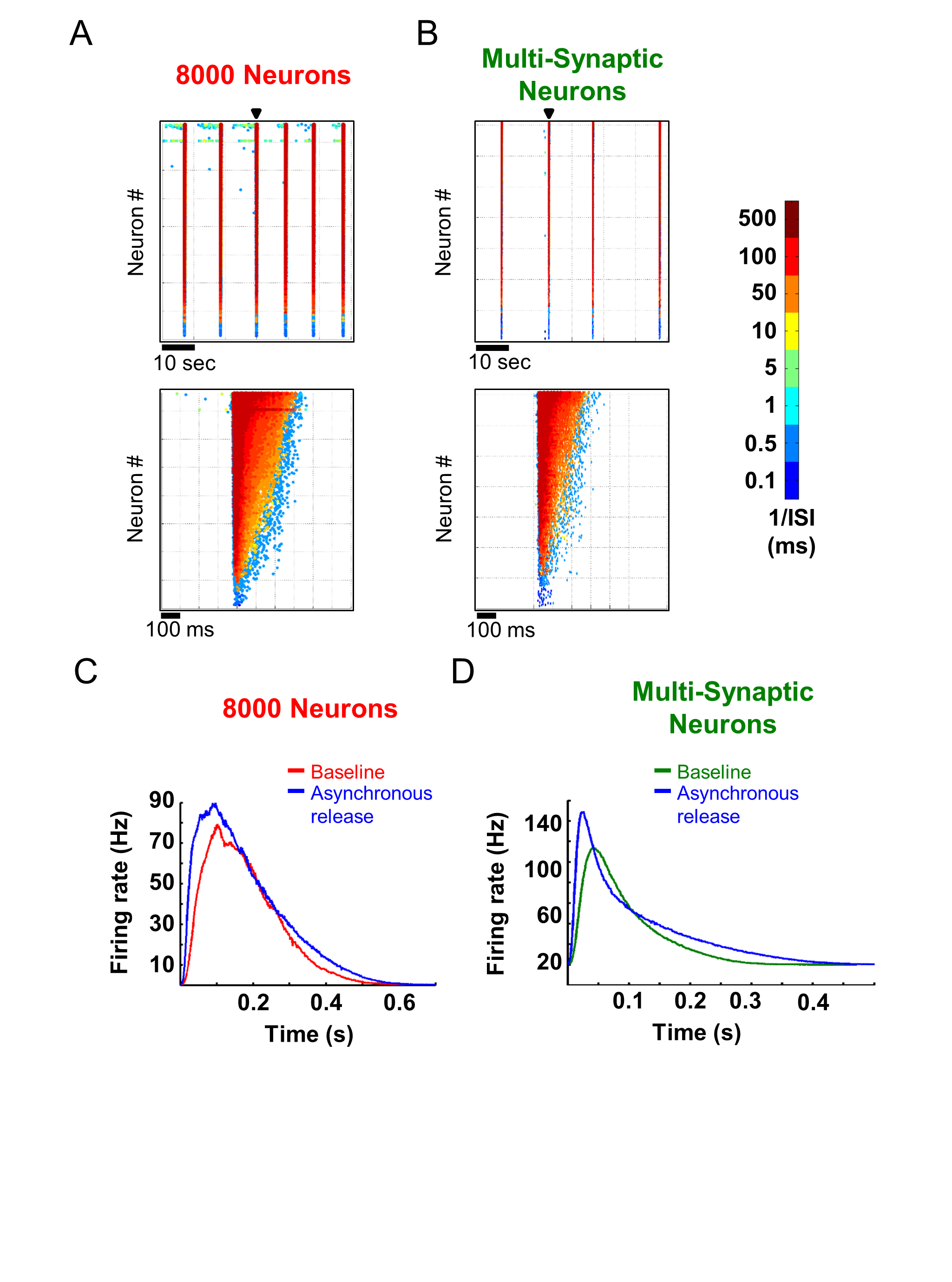


**Figure S5. Primary effects of asynchronous release on network activity are maintained following substantial changes to network structure.** (**A**) Raster plot of spontaneous activity of a simulated neuronal network with a 10-fold increase in the number of neurons (8000 neurons, top panel; lower panel displays a representative network burst marked by arrow in the upper panel). (**B**) Raster plot of spontaneous activity of a simulated neuronal network with a 10-fold increase in the number of synapses per neuron (10 independent synapses per neuron, top panel; lower panel displays a representative network burst marked by arrow in the upper panel). Under a 10-fold increase in the number of neurons (**C**) or 10-fold increase in the number of synapses per neuron (**D**), the enhanced asynchronous release still increases peak network burst firing rate and reduces the network burst time to peak.
