# Supplementary material for: Shaping Neuronal Network Activity by Presynaptic Mechanisms
Source: PLoS Comput Biol. 2015 Sep 15;11(9):e1004438. doi: 10.1371/journal.pcbi.1004438 (PMC4570815; doi:10.1371/journal.pcbi.1004438)
Supplement: S6 Fig — (A) Higher asynchronous release following DOC2B overexpression and strontium application significantly increases the ratio of "full" bursts, while higher spontaneous release following DOC2BD218,220N decreases this ratio compared to GFP control cultures (top panel; *P < 0.05, **P < 0.01, under one-way ANOVA; error bars show SEM; modified from Lavi et al. [3]). In the model, analysis of the "full" vs. "aborted" bursts follows the experimental results (bottom panel; ***P < 0.001 under one-way ANOVA; error bars show SEM); higher spontaneous release in the model significantly decreases the ratio of "full" bursts while the increase in asynchronous release in the model increases the same ratio. (B) Network burst synchronization analysis shows that enhanced asynchronous release, induced by DOC2B overexpression or strontium application, significantly increases network burst synchronization, while higher spontaneous release frequency following DOC2BD218,220N overexpression significantly reduces network burst synchronization (top panel; each line represents the average change from baseline conditions in pairwise Pearson correlation for all active electrodes in the network burst; 15 DOC2B recordings, 6 strontium recordings, 9 DOC2BD218,220N recordings; *P < 0.05, **P < 0.01, ***P < 0.001, ANOVA for repeated measurements; error bars show SEM; modified from Lavi et al. [3]). In agreement with the experimental findings, analysis of network burst synchronization in the model shows that while spontaneous release significantly reduces network burst peak synchronization, asynchronous release significantly increases peak network burst synchronization (bottom panel; *P < 0.05, **P < 0.01, ***P < 0.001, ANOVA for repeated measurements; error bars show SEM). The increase in the model is most significant during the peaks of the bursts and seems to be shorter than the experimental effect, perhaps due to the reduced variability in the model's burst duration. (DOCX) [file pcbi.1004438.s006.docx]

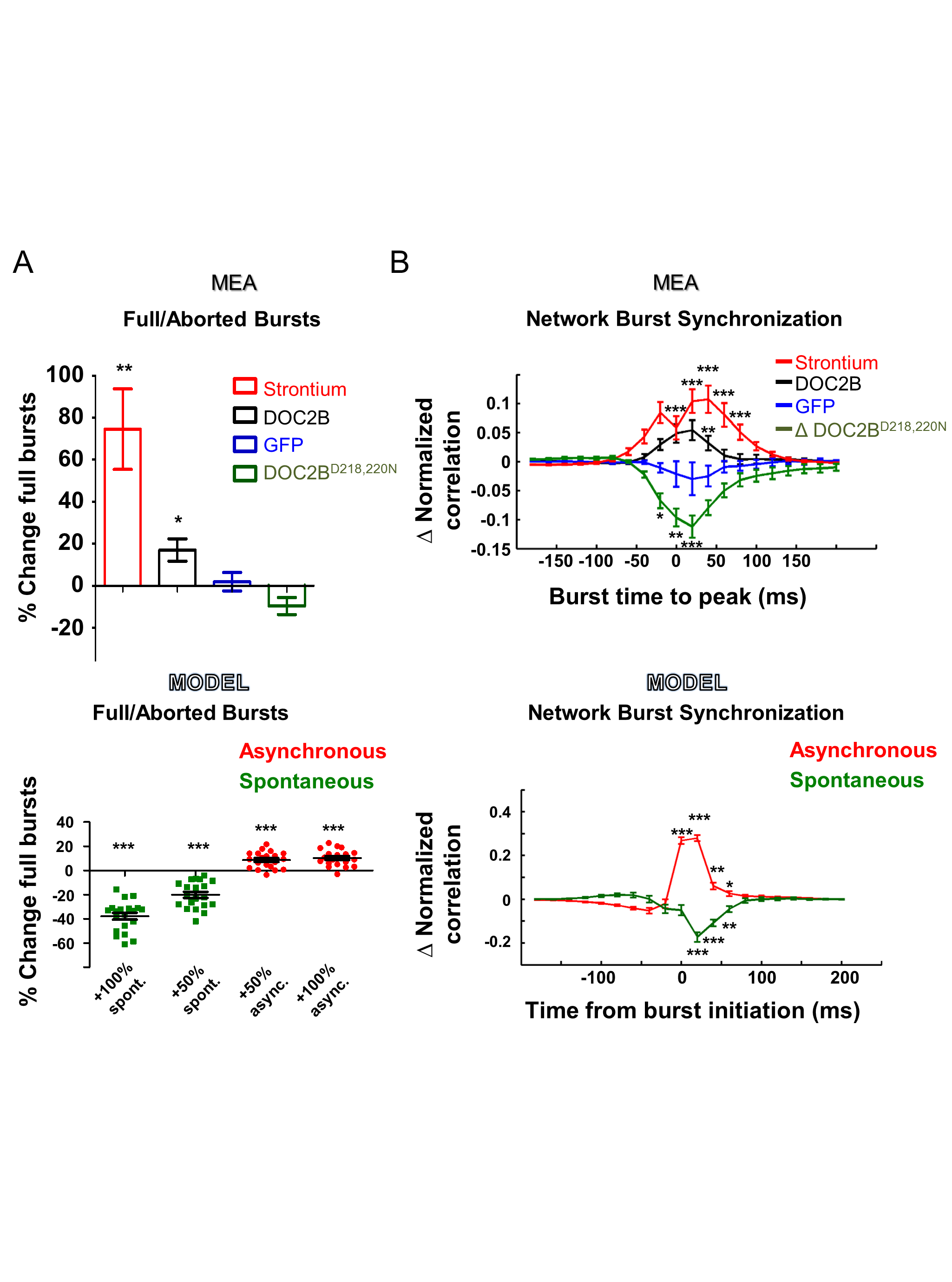


**Figure S6. Enhanced asynchronous release increases network synchronization and percentage of "full" bursts.** **(A)** Higher asynchronous release following DOC2B overexpression and strontium application significantly increases the ratio of "full" bursts, while higher spontaneous release following DOC2B^D218,220N^ decreases this ratio compared to GFP control cultures (top panel; **P* < 0.05, ***P* < 0.01, under one-way ANOVA; error bars show SEM; modified from Lavi et al. [3]). In the model, analysis of the "full" vs. "aborted" bursts follows the experimental results (bottom panel; ****P* < 0.001 under one-way ANOVA; error bars show SEM); higher spontaneous release in the model significantly decreases the ratio of "full" bursts while the increase in asynchronous release in the model increases the same ratio. **(B)** Network burst synchronization analysis shows that enhanced asynchronous release, induced by DOC2B overexpression or strontium application, significantly increases network burst synchronization, while higher spontaneous release frequency following DOC2B^D218,220N^ overexpression significantly reduces network burst synchronization (top panel; each line represents the average change from baseline conditions in pairwise Pearson correlation for all active electrodes in the network burst; 15 DOC2B recordings, 6 strontium recordings, 9 DOC2B^D218,220N^ recordings; **P* < 0.05, ***P* < 0.01, ****P* < 0.001, ANOVA for repeated measurements; error bars show SEM; modified from Lavi et al. [3]). In agreement with the experimental findings, analysis of network burst synchronization in the model shows that while spontaneous release significantly reduces network burst peak synchronization, asynchronous release significantly increases peak network burst synchronization (bottom panel; **P* < 0.05, ***P* < 0.01, ****P* < 0.001, ANOVA for repeated measurements; error bars show SEM). The increase in the model is most significant during the peaks of the bursts and seems to be shorter than the experimental effect, perhaps due to the reduced variability in the model's burst duration.
